# Structural insights into the agonists binding and receptor selectivity of human histamine H_4_ receptor

**DOI:** 10.1038/s41467-023-42260-z

**Published:** 2023-10-20

**Authors:** Dohyun Im, Jun-ichi Kishikawa, Yuki Shiimura, Hiromi Hisano, Akane Ito, Yoko Fujita-Fujiharu, Yukihiko Sugita, Takeshi Noda, Takayuki Kato, Hidetsugu Asada, So Iwata

**Affiliations:** 1 Department of Cell Biology, Graduate School of Medicine, Kyoto University, Konoe-cho, Yoshida, Sakyo-ku, Kyoto, 606-8501 Japan; 2 Institute for Protein Research, Osaka University, 3-2 Yamadaoka, Suita, Osaka, 565-0871 Japan; 3 Institute of Life Science, Kurume University, Kurume, Fukuoka 830-0011 Japan; 4 Laboratory of Ultrastructural Virology, Institute for Life and Medical Sciences, Kyoto University, 53 Shogoin Kawahara-cho, Sakyo-ku, Kyoto, 606-8507 Japan; 5 Laboratory of Ultrastructural Virology, Graduate School of Biostudies, Kyoto University, 53 Shogoin Kawahara-cho, Sakyo-ku, Kyoto, 606-8507 Japan; 6 CREST, Japan Science and Technology Agency, 4-1-8 Honcho, Kawaguchi, Saitama 332-0012 Japan; 7 Hakubi Center for Advanced Research, Kyoto University, Kyoto, 606-8501 Japan; 8 RIKEN SPring-8 Center, 1-1-1 Kouto, Sayo-cho, Sayo-gun, Hyogo, 679-5148 Japan

**Keywords:** Cryoelectron microscopy, Cell signalling, G protein-coupled receptors

## Abstract

Histamine is a biogenic amine that participates in allergic and inflammatory processes by stimulating histamine receptors. The histamine H_4_ receptor (H_4_R) is a potential therapeutic target for chronic inflammatory diseases such as asthma and atopic dermatitis. Here, we show the cryo-electron microscopy structures of the H_4_R-G_q_ complex bound with an endogenous agonist histamine or the selective agonist imetit bound in the orthosteric binding pocket. The structures demonstrate binding mode of histamine agonists and that the subtype-selective agonist binding causes conformational changes in Phe344^7.39^, which, in turn, form the “aromatic slot”. The results provide insights into the molecular underpinnings of the agonism of H_4_R and subtype selectivity of histamine receptors, and show that the H_4_R structures may be valuable in rational drug design of drugs targeting the H_4_R.

## Abstract (web summary)

Histamine receptor H_4_R is implicated in chronic inflammatory diseases, such as asthma, arthritis, and atopic dermatitis. Here, the authors determined the first cryo-EM structure of the histamine H4 receptor, providing valuable structural insights for the design of drugs targeting H4R in chronic inflammatory diseases.

## Introduction

Histamine, a biogenic neurotransmitter, exerts its pathophysiological functions in the central nervous system and peripheral tissues by stimulating histamine receptors belonging to the G protein-coupled receptor (GPCR) superfamily. Four histamine receptors (H_1_R–H_4_R) have been identified in humans, each with a different intracellular signaling mechanism^1^. In 1988 a Nobel prize was awarded for the discovery of H_2_R antagonists, following the pharmacological discovery of H_1_R and H_2_R^2,3^. The antagonists of these receptors have been developed for the treatment of allergic diseases and gastric acid secretion, respectively, and are still in use^1,4^. H_3_R was identified in the 80 s and has very low homology (<20%) with H_1_R and H_2_R^5,6^. It is a clinical target for central nervous system diseases, as it mainly regulates histamine function in the brain, and the H_3_R antagonist pitolisant has been used for the treatment of narcolepsy (Wakix®) and obstructive sleep apnea (Ozawade®)^7^.

H_4_R is the last histamine receptor to be identified^8–12^. Since H_4_R is mainly expressed in hematopoietic cells, such as monocytes, basophils, dendritic cells, and T-lymphocytes, it has been reported to participate in the release of cytokines, and consequently plays an important role in anti-inflammatory and immunomodulatory activities^13,14^. Therefore, H_4_R may be a target for chronic inflammatory diseases (CIDs), such as asthma, arthritis, and atopic dermatitis^15,16^. Preclinical and clinical studies involving H_4_R antagonists have demonstrated its therapeutic efficacy against CIDs, making H_4_R a promising therapeutic target^4^. H_4_R shares 34% of its amino acid sequence identity with H_3_R, and 50% in the transmembrane (TM) region. Because of their structural similarities, there are many ligands that have a high affinity for both receptors^17,18^. However, it is important to further explore selective H_4_R ligands to facilitate the development of more effective CID therapies.

In a previous study, we determined the crystal structure of the inactive form of H_1_R (H_1_R_dox_), which is bound to doxepin, a first-generation antihistamine^19^. In the earlier study, the binding mode of doxepin and the reason for the low selectivity of first-generation antihistamines were investigated. Furthermore, the binding mode and specificity of second-generation antihistamines were determined using a computational approach. Recently, the cryo-electron microscopy (cryo-EM) structure of active H_1_R (H_1_R_his_) bound to an endogenous agonist histamine has been reported^20^. The structure revealed details of histamine binding in H_1_R. The activation mechanism of H_1_R was assessed further by examining the details of G_q_ protein engagement.

Here, we report the cryo-EM structures of H_4_R, which include H_4_R bound to an endogenous histamine ligand (H_4_R_his_) and H_4_R bound to the H_3_R/H_4_R selective agonist imetit (H_4_R_ime_). We elucidate the specific ligand recognition mechanism of H_4_R by comparing the binding mode of two agonists with different pharmacological characteristics. Furthermore, these results may provide insights that facilitate rational drug design targeting the H_4_R.

## Results

### Overall structures of the agonist-bound H_4_R-G_q_ complex

For the cryo-EM experiment, H_4_R and trimeric G proteins (Gα_q_, rat Gβ_1_, and bovine Gγ_2_) were co-expressed using *Spodoptera frugiperda* (Sf9) insect cells. H_4_R is known to primarily engage with Gα_i_ and transduce G_i_ signals. We co-expressed and purified G_i_-coupled H_4_R but failed to generate the complex (Supplementary Fig. 1b). However, the complex was successfully obtained by using the non-canonical miniG_q_iN as an accessory protein for structural determination. Furthermore, H_4_R has been reported to have a weak G_q_ signal (pEC_50_ = 6.8, Emax = 9% for G_q_/pEC_50_ = 7.1, Emax = 26% for G_i_)^21^; therefore, the H_4_R-G_q_ complex was used to determine the H_4_R structure.

**Fig. 1:**
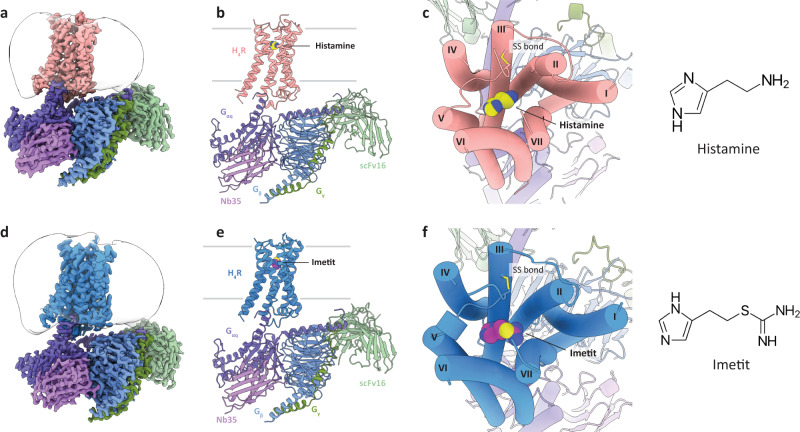
Overall structures of the H_4_R signaling complexes. **a**, **b** Orthogonal views of the cryo-EM density map (**a**) and model (**b**) of the histamine-bound H_4_R-G_q_ complex. **c** Extracellular view of the histamine-bound H_4_R-G_q_ complex and chemical structure of histamine. **d**, **e** Orthogonal view of the cryo-EM density map (**d**) and model (**e**) of the imetit-bound H_4_R-G_q_ complex. **f** Extracellular view of the imetit-bound H_4_R-G_q_ complex and chemical structure of imetit. Cys87^3.25^ and Cys164^ECL2^ form a disulfide bond and are shown as stick models (**c**, **f**). In (**a**)–(**f**), the complexes are colored by subunits. Histamine-bound H_4_R: pink, imetit-bound H_4_R: blue, G_q_: purple, Gβ: navy, Gγ: green, Nb35: violet, scFv16: lime, histamine: yellow, and imetit: magenta.

The H_4_R-G_q_ complex structures, H_4_R_his_ and H_4_R_ime_, which were bound to histamine and imetit, were determined by a cryo-EM single-particle analysis at 3.0 Å and 3.1 Å resolutions, respectively (Fig. 1, Supplementary Fig. 2, and Table 1). In the H_4_R_his_ and H_4_R_ime_ structures, the side chains of most of the amino acids in the receptor and G protein regions could be assigned to the final EM map and refined with excellent geometry. Both the agonists used in the present study, histamine and imetit, were clearly identified, and a model was obtained to explain the recognition mechanisms of the agonists in H_4_R (Supplementary Fig. 3). In the overall structure of the H_4_R-G_q_ complex, similar to other GPCR-G protein complex structures, the G_q_ trimer bound to the intracellular region of H_4_R, and Nb35^22^ and scFv16^23^ bound to G_q_ and the G_q_-Gβ interface, respectively, thereby stabilizing the complex (Fig. 1a, b, d, e). A local resolution analysis showed that the WD40 repeat core of the Gβ subunit, N-terminal region of the G_α_ subunit (G_α_HN), and scFv16 had the highest resolution, whereas the extracellular region of the receptor and a portion of the N-terminal region of Gβγ had a lower resolution (Supplementary Figs. 2e, f). In addition, the N/C-terminus, the intracellular loop 3 (ICL3), and some parts of the extracellular loops (ECL2, ECL3) of H_4_R were difficult to observe due to their high flexibility, and therefore, could not be modeled.

**Table 1:** Cryo-EM data collection, refinement, and validation statistics

	Histamine-H_4_R-G_q_ complex (EMDB-33785) (PDB 7YFC)	Imetit-H_4_R-G_q_ complex (EMDB-33786) (PDB 7YFD)
**Data collection and processing**		
Magnification	81,000	81,000
Voltage (kV)	300	300
Electron exposure (e–/Å^2^)	50	50
Defocus range (μm)	−0.8 to −2.0	−0.8 to −2.0
Pixel size (Å)	0.88	0.88
Symmetry imposed	C1	C1
Initial particle images (no.)	1,823,691	2,410,519
Final particle images (no.)	180,728	628,467
Map resolution (Å)	3.0	3.1
FSC threshold	0.143	0.143
**Refinement**		
Initial model used (PDB code)	7DFL, 7F9Z	7DFL, 7F9Z
Model resolution (Å)	3.3	3.2
FSC threshold	0.5	0.5
Map sharpening *B* factor (Å^2^)	−108.22	−134.07
Model composition		
Non-hydrogen atoms	9882	9934
Protein residues	1262	1260
Ligands	HSM; 1, CLR: 1	IME: 1, CLR: 2
*B* factors (Å^2^)		
Protein	58.9	37.7
Ligand	64.3	64.9
R.m.s. deviations		
Bond lengths (Å)	0.005	0.003
Bond angles (°)	0.656	0.534
Validation		
MolProbity score	1.44	1.57
Clashscore	5.92	7.16
Poor rotamers (%)	0.09	0.19
Ramachandran plot		
Favored (%)	97.42	97.01
Allowed (%)	2.58	2.99
Disallowed (%)	0	0

The H_4_R_his_ and H_4_R_ime_ structures exhibited a canonical GPCR scaffold with seven transmembrane helices (TM1–7) and an intracellular amphipathic helix 8 (H8) (Fig. 1c, f, Supplementary Fig. 3c). The root mean square deviation of both structures was 0.32 Å for the overall structure and 0.60 Å for the receptor region, indicating similar conformations. Histamine and imetit occupied an orthosteric binding pocket in the middle of the TMs, which was covered by a C-terminal segment of ECL2 stabilized by the disulfide bonds of C87^3.25^ and C164^ECL2^, which are common in other class A GPCRs (Fig. 1c, f).

### Molecular basis for the recognition of histamine and imetit by H_4_R

**Fig. 2:**
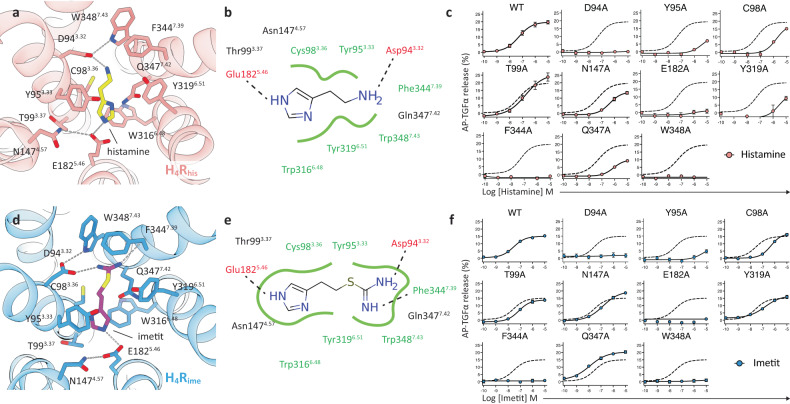
Agonist recognition of H_4_R. **a** Detailed interactions between histamine and H_4_R. Histamine and contact residues are shown as yellow and pink sticks. **b** Schematic representation of histamine-binding interactions. Hydrogen bonds are shown as dashed lines, and hydrophobic interactions and amino acids are shown in green. **c** Mutant study to assess the histamine response by the TGFα shedding assay. Dashed lines in the mutant graphs represent the wild-type (WT) H_4_R response. Concentration-response curves are shown as the mean ± standard error of the mean (SEM) from three independent experiments performed in triplicates. In many data points, the error bars are smaller than the symbols and are therefore not visible. **d** Detailed interactions between imetit and H_4_R. Imetit and contact residues are shown as magenta and blue sticks. **e** Schematic representation of imetit-binding interactions. **f** Mutant study to assess the imetit response by the TGFα shedding assay. Details are the same as in (**c**).

Histamine and imetit share a common imidazole backbone, and imetit is characterized by a more extended structure with an additional isothiourea group, when compared with histamine (Fig. 1c, f). In the cryo-EM structure, histamine and imetit shared a common ligand-binding pocket surrounded by residues consisting of TM3, 4, 5, 6, and 7 (Fig. 2a, b, d, e). The binding mode of histamine and imetit was verified by a transforming growth factor (TGF)-α shedding assay using mutants of H_4_R, and was observed to be consistent with that in the model structure (Fig. 2c, f, Supplementary Fig. 4 and Supplementary Table 1)^24^.

In the structure of H_4_R_his_, the primary amino group of the ethylamine portion of histamine formed a salt bridge with Asp94^3.32^ (Fig. 2a). This interaction is widely conserved in aminergic receptor structures, including the H_1_R structure. A loss of activity was observed in the D94^3.32^A mutant of H_4_R_his_ (Fig. 2c). In many aminergic receptors, Asp^3.32^ forms a hydrogen bond with the neighboring Trp/Tyr^7.43^, thereby stabilizing its side chain and facilitating interactions with the amine group of the ligand^25^. Hydrogen bonding between Asp94^3.32^ and Trp348^7.43^ was also observed in H_4_R_his_, and the loss of signaling activity of the mutant W348^7.43^A indicated that the stability of Asp94^3.32^ is important for ligand binding (Fig. 2a, c). The TM7 residues of Phe344^7.39^, Gln347^7.42^, and Trp348^7.43^ are located within the van der Waals radius of the primary amino group of histamine and form hydrophobic interactions with it. Furthermore, Gln347^7.42^ forms weak polar interactions with the imidazole group of histamine. These interactions are essential for histamine binding, and their importance was confirmed by the reduced activity of Phe344^7.39^ and Gln347^7.42^ in the Ala mutant (Fig. 2c).

On the opposite side of histamine from Asp94^3.32^, Tyr319^6.51^ is conserved in aminergic receptors in a similar manner as Tyr/Phe^6.51^, suggesting that it is an essential residue for agonist and antagonist binding^19,20,26,27^. In H_4_R_his_, histamine forms hydrophobic contacts with this residue, and it was verified that the introduction of mutations to this residue is directly related to the decrease in receptor activity (Fig. 2a–c). Tyr/Phe^6.51^ of TM6 plays an important role in ligand binding by forming aromatic clusters with Trp^6.48^ and Phe^6.52^ in the aminergic receptor^28^. In addition to ionic binding to Asp^3.32^, many aminergic ligands form an aromatic stacking or hydrophobic interaction with this aromatic cluster. In H_4_R_his_, Trp316^6.48^ was further coordinated to the bottom of the ligand binding pocket and exhibited a stacking interaction with the imidazole ring of histamine. So far, H_4_R mutant experiments have already been conducted on some of the above-mentioned amino acid residues in previous studies^29,30^, and it was confirmed that there was no significant deviation from the experiments in the present study.

The imidazole ring of histamine was bound to a pocket consisting of Tyr95^3.33^, Thr99^3.37^, Asn147^4.57^, Glu182^5.46^, Trp316^6.48^, and Tyr319^6.51^. As mentioned above, the imidazole ring was stabilized by pi–pi stacking interactions from both the upper and lower sides, with Tyr95^3.33^ as a lid, in addition to Trp316^6.48^ at the bottom of the pocket (Fig. 2a, b). In particular, the Y95^3.33^A mutant dramatically reduced histamine activity, suggesting that it plays an essential role in binding (Fig. 2c). In addition, Glu182^5.46^ is an important residue in the binding of the imidazole ring. The nitrogen atom (N^τ^) on the third position of the imidazole ring formed an ionic pair with Glu182^5.46^, suggesting the importance of Glu182^5.46^ along with that of Asp94^3.32^ in the binding process. Previous studies have reported that mutants of this residue prevent histamine binding. This is in line with our signaling activity data (Fig. 2c)^29,30^. Although Thr99^3.37^ and Asn147^4.57^, which form a pocket, do not interact directly with the ligand, the signaling activity of the T99A^3.37^ and N147A^4.57^ mutants was found to be 2.4- and 9.2-fold lower, respectively, than that of wild-type H_4_R. Thr99^3.37^ is involved in pocket formation, and Asn147^4.57^ contributes to the stabilization of the interaction with histamine by forming a hydrogen bond with the neighboring Glu182^5.46^ and regulating the coordination of its side chain, as observed between Trp348^7.43^ and Asp94^3.32^ (Fig. 2a–c, Supplementary Table 1). Position 4.57 is Asn^4.57^ in human H_4_R but His^4.57^ in pigs and dogs; therefore, altering the side chain conformation of Glu^5.46^ has been reported to be a possible reason for the species-specific differences in histamine affinity^31^.

In H_4_R_ime_, imetit was found to bind to a pocket in a manner similar to that of histamine (Fig. 2a, b, d, e). Imetit had two hydrogen bond donors, which were complementary to the two negative residues of the binding pocket, Asp94^3.32^ and Glu182^5.46^, respectively. Mutagenesis experiments confirmed that these interactions are essential for imetit binding (Fig. 2f). Previous studies have suggested that the isothiourea group is a key determinant of the H_4_R activity enhancement in histaminergic compounds^18^. The cryo-EM structure of H_4_R_ime_ revealed that the isothiourea group of imetit plays a significant role in ligand binding. This substructure of the agonist penetrated the subpocket that is formed by mostly aromatic and hydrophilic residues, such as Tyr319^6.51^, Phe344^7.39^, Gln347^7.42^, and Trp348^7.43^ (Fig. 2d, e). The two nitrogen atoms existing in the region formed a salt bridge with Asp94^3.32^ for the primary amino group and a hydrogen bond with the oxygen atom of the main chain of Phe344^7.39^ for the methanimine group. The primary amino group not only interacted with Asp94^3.32^, but also formed pi–cation interactions with Tyr319^6.51^, Phe344^7.39^, and Trp348^7.43^, which constitute the hydrophobic pocket, thereby increasing ligand binding stability. Mutant Y319A^6.51^ exhibited a 5.3-fold decrease in signaling, while mutants F344A^7.39^ and W348A^7.43^ had even greater effects when compared with wild-type H_4_R, indicating that the residues are crucial for imetit binding (Fig. 2f, Supplementary Table 1). The imidazole ring of imetit was tightly accommodated in a pocket consisting of TM3, 4, 5, and 6 (Fig. 2d, e). Furthermore, the hydrophobic interactions formed between imetit and Tyr95^3.33^, Cys98^3.36^, Thr99^3.37^, and Asn147^4.57^ were shown to be crucial for its binding (Fig. 2f, Supplementary Table 1). Similar to histamine, the imidazole ring of imetit was stably coordinated in the pocket by forming stacking hydrophobic interactions with Tyr95^3.33^ and Trp316^6.48^. However, unlike histamine, the imidazole group of imetit does not interact with Gln347^7.42^. This may be the difference between the two agonists in the Q347A^7.42^ mutant (Fig. 2c, f, Supplementary Fig. 4b).

### Comparison of agonist-binding characteristics between H_4_R_his_ and H_4_R_ime_

**Fig. 3:**
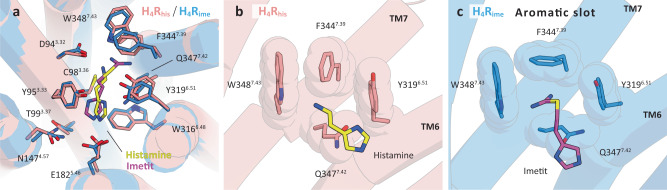
Comparison of the binding poses between histamine and imetit for H_4_R. **a** Structural alignment of the agonist-binding pocket of H_4_R_his_ (pink) and H_4_R_ime_ (blue). Histamine (yellow), imetit (magenta), and the surrounding residues of each agonist are shown as sticks. **b**, **c** Hydrophobic residue cluster of TM7 and 8 in H_4_R_his_ (**b**) and H_4_R_ime_ (**c**). The side chain rearrangement of Phe344^7.39^ forms the aromatic slot on H_4_R_ime_. Residues comprising the pocket are indicated by sticks and transparent spheres and are pink (H_4_R_his_) and blue (H_4_R_ime_).

Imetit was initially developed as an agonist for H_3_R^32,33^ but has also been reported to have a high binding affinity (K_i_) for H_4_R (imetit K_i_ = 2.7 nM, histamine K_i_ = 8.1 nM)^10^. As described in the previous section, the residues constituting the orthosteric binding site in H_4_R_his_ and H_4_R_ime_ were almost identical. However, the binding mode of the two agonists was clearly different (Fig. 3, Supplementary Figs. 5a, b). Imetit has a larger surface area than histamine due to the substituted isothiourea moiety (histamine: 267.8 Å^2^ vs. imetit: 372.3 Å^2^) (Supplementary Fig. 5c). Although H_4_R_his_ and H_4_R_ime_ possess ligand-binding pockets of approximately the same size, several unique recognition mechanisms were established to make each agonist acceptable. First, remarkable differences in the binding features of both agonists were observed in the pockets around TM6 and 7. The H_4_R_ime_ structure formed a distinct pocket (hereafter referred to as the “aromatic slot”) consisting of Tyr319^6.51^, Phe344^7.39^, Trp348^7.43^, and Gln347^7.42^, which was not observed in the H_4_R_his_ structure. Compared to the histamine-bound structure, this pocket was generated by the Cβ–Cγ bond of Phe344^7.39^ that rotated approximately 90 degrees, like a turning doorknob, allowing it to accept a more extended imetit (Fig. 3b, c). The isothiourea moiety of imetit is likely protonated and charged. Both nitrogen atoms are equivalent and actively utilize this specific pocket to form multiple interactions with residues in the pocket. This may contribute to the increased binding strength of imetit. In contrast, the primary amine of histamine interacts relatively simply with Asp94^3.32^ and Phe344^7.39^, while the isothiourea portion of imetit utilizes the entire aromatic slot, which may be one of the reasons why the binding affinity of imetit is approximately three times higher than that of histamine^10^.

The interaction between imetit and the aromatic slot and the overall molecular geometry of imetit caused its imidazole ring to coordinate 1.8 Å closer to TM3 than histamine. Imetit further exhibited a stronger interaction with peripheral residues such as Cys98^3.36^ and Thr99^3.37^, which may have also contributed to the binding affinity of imetit for H_4_R (Fig. 3a). The formation of a distinct ligand-specific binding pocket by the conformational change of the Phe side chain, as observed in the aromatic slot of H_4_R, has also been reported for the extended binding pocket in the crystal structure of the dopamine D_2_ receptor (in this case, Phe^3.28^), which is a similar aminergic receptor^27^. Since it is difficult to consider the structural plasticity of ligand-binding pockets in drug design, identifying the flexible ligand-binding pocket shown in our structure may make a significant contribution to rational drug design.

### Subtype-selectivity of H_4_R and H_1_R

While the affinity of H_1_R and H_2_R for histamine is in the μM range, H_3_R and H_4_R are known to have a high affinity in nM range^1,34^. Histamine receptor subtypes therefore require different concentrations of histamine for activation; however, the molecular basis for the selectivity between these subtypes remains unknown given the lack of structural information on histamine receptors. Here, we assessed the selectivity between subtypes in histamine receptors by comparing the cryo-EM structure H_1_R_his_ of histamine-bound H_1_R^20^ with the structures H_4_R_his_ and H_4_R_ime_ obtained in the present study.

**Fig. 4:**
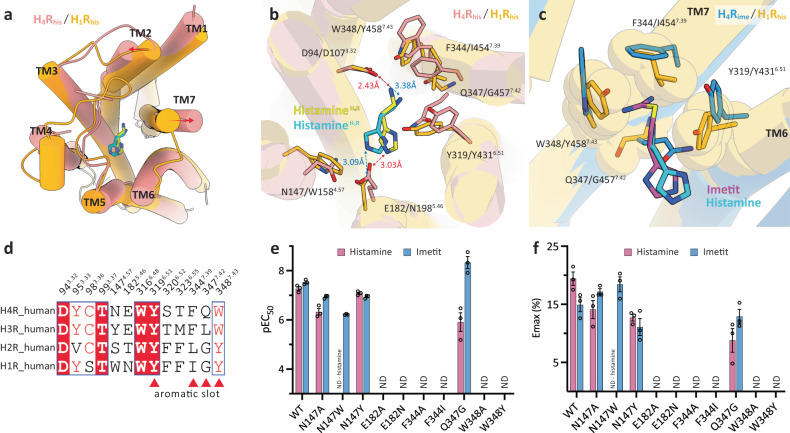
Subtype selectivity in H_4_R and H_1_R. **a**, **b** Structural superposition of H_4_R_his_ (pink) and H_1_R_his_ (orange, PDB 7DFL). (**a**) top view, (**b**) the agonist-binding pocket. The helices are shown as cylinders. The histamines of H_4_R and H_1_R are indicated by yellow and cyan sticks. **c** The aromatic slot on H_4_R_ime_ (blue) and the corresponding region for H_1_R_his_ (orange). Imetit and histamine are shown as magenta and cyan sticks. Residues comprising the pocket for H_4_R_ime_ are indicated by sticks and for H_1_R_his_ are shown as sticks and transparent spheres. **d** Sequence alignment of residues important for agonist binding in the histamine receptor family. Red triangles indicate residues corresponding to the aromatic slots in H_4_R. **e**, **f** TGFα shedding assay results of WT H_4_R and H_4_R mutants activated by histamine and imetit. The activities of the agonists are identified as pEC50 (**e**). Average Emax values were determined from the “log (agonist) vs. response -variable slope (three or four parameters)” function in GraphPad Prism 9 software (GraphPad Software Inc., San Diego, CA) (**f**). Data represent the mean ± SEM from *n* = 3 biologically independent experiments performed in triplicate. ND, not detected.

The overall structure of the receptor region of H_1_R_his_ and H_4_R_his_ was relatively similar except for the extracellular ends of TM2 and 7 and the positions of the orthosteric binding pocket to which histamine bonds were almost identical (Fig. 4a). However, some of the residues that formed the ligand-binding pocket were different, which may have determined the affinity of histamine for both receptors. The most significant difference between the two receptors in histamine binding was the amino acid corresponding to position 5.46. Asn192^5.46^ of H_1_R_his_ and Glu182^5.46^ of H_4_R_his_ formed hydrogen bonds with N^τ^ atoms in the imidazole ring of histamine; however, in H_4_R, histamine exerted a stronger interaction with negatively charged Glu182^5.46^ by establishing an ionic association (Fig. 4b). In contrast, the corresponding amino acid Asn192^5.46^ in H_1_R is a neutral amino acid; therefore, the interaction was relatively weak, and the binding energy may have been lower than that in H_4_R. As described in the previous section, Glu182^5.46^ in H_4_R had its side chain coordination stabilized by hydrogen bonding with the neighboring Asn147^4.57^, whereas position 4.57 in H_1_R was Trp and could not form a hydrogen bond network with position 5.46. The side chain stabilization effect was therefore not achieved. These structural differences were thought to affect the stability of the interaction with the N^τ^ atom of histamine. In fact, the H_1_R mimic mutants E182N^5.46^ and N147W^4.57^ showed a loss of signaling activity for histamine, while the H_3_R type mutant N147Y^4.57^ at position 4.57 exhibited relatively similar EC_50_ compared to wild type H_4_R (WT 55.4 nM vs. mutant 82.9 nM), as verified by the TGFα shedding assay. This indicated that the configuration and interaction of the residues in positions 4.57 and 5.46 in histamine binding are some of key factors that influence the difference in affinity between H_1_R and H_4_R (Fig. 4e, f, Supplementary Fig. 4 and Supplementary Table 1).

The primary amino group in histamine also exhibited different binding for both receptors. The ionic interaction at position 3.32, which is common in aminergic receptors, was conserved in both H_1_R_his_ and H_4_R_his_. In H_1_R_his_, however, the cationic amine moiety of histamine interacted relatively simply with Asp107^3.32^ and Tyr431^6.51^, whereas in H_4_R_his_, it interacted with the hydrophobic residue cluster on TM7 to gain greater binding affinity (Fig. 4b). Position 7.39 differed between H_1_R and H_4_R based on Ile and Phe, respectively, and comprised Phe344^7.39^ in H_4_R, which has a bulkier side chain that was present within a distance where it could interact with histamine. The H_1_R mimic mutant F344I^7.39^ did not exhibit histamine activity, which indicated that this residue plays a critical role in histamine recognition (Fig. 4e, f). We posit that the interaction between histamine and Gln347^7.42^ also affects histamine affinity. As previously mentioned, in the H_4_R_his_, Gln347^7.42^ forms a polar interaction with the imidazole ring of histamine. However, such binding is not observed in H_1_R_his_ and the signaling activity of the H_1_R/H_2_R type mutant Q347G^7.42^ decreases about 46 times compared to that of the wild type (Fig. 4e, f, Supplementary Fig. 4 and Supplementary Table 1). Based on such observations, we consider the interaction of Gln347^7.42^ and histamine in H_4_R to be one of the reasons for the high affinity.

Imetit is an H_3_R/H_4_R selective agonist that does not bind to H_1_R/H_2_R and has a high binding affinity for H_3_R/H_4_R of 0.3 and 2.7 nM, respectively^10,33^. Since the publication of the structure of the first histamine receptor, doxepin-bound human H_1_R^19^, various computational chemistry approaches have been used to determine the molecular determinants of H_3_R/H_4_R ligand binding, and several binding modes have been predicted. However, in most reports, no determinant other than Glu182^5.46^ has been found to be important for subtype selectivity^35,36^. In the present study, we determined the structure of imetit-bound H_4_R, which can be compared with H_1_R_his_, to assess the mechanisms underlying subtype selectivity in histamine receptors. As mentioned above, a detailed investigation of histamine binding to H_1_R/H_4_R revealed that Glu182^5.46^ is undoubtedly one of the key residues involved in the selectivity of histamine receptors (Fig. 4b–f). Moreover, we identified the aromatic slot, a pocket that is crucial for the binding of the isothiourea region of imetit, as an important region for the selectivity of H_1_R and H_4_R (Fig. 4c). The aromatic slot consisting of Tyr319^6.51^, Phe344^7.39^, Gln347^7.42^, and Trp348^7.43^ was formed by the flexible orientation of the side chain of Phe in position 7.39, which is characterized by a mutually reinforced shape (Fig. 3b, c). The most important factor for the correct function of the aromatic slot is the flexibility of the side chain at position 7.39. In H_3_R/H_4_R, the residues corresponding to this position are both Phe, but in H_1_R/H_2_R, they are Ile and Leu, respectively. The formation and maintenance of the aromatic slot by conformational changes in these side chains are therefore unlikely (Fig. 4c, d). Furthermore, the orientations of Ile454^7.39^ and Tyr458^7.43^ in H_1_R are likely to cause steric hindrance with the two nitrogen atoms in the isothiourea group. The acceptance of the isothiourea group of imetit in H_1_R is further likely to be challenging given the restricted coordination of the Tyr458^7.43^ side chain in maintaining the Asp^3.32^–Trp/Tyr^7.43^ interaction that is common in aminergic receptors.

The importance of the aromatic slot in the selectivity of histamine receptors was also verified in mutant experiments. H_1_R mimic mutants F344I^7.39^ and W348Y^7.43^ exhibited a loss of imetit activity, thereby supporting the above structural findings (Fig. 4e, f). However, Q347G^7.42^ exhibited a slight increase in the imetit signal, which is consistent with the activity for Q347A^7.42^ mutant mentioned earlier. The conformation of the extended side chain of Gln347^7.42^ is thought to restrict the conformation of Trp316^6.48^, and the removal of this restriction by mutation of Gln347^7.42^ gives the Trp316^6.48^ rotamer more flexibility, suggesting that it may interact more closely with imetit. Since the interaction between Gln347^7.42^ and the imidazole ring of histamine was observed, we believe that its activity is reduced by the same mutations (Fig. 4e, f). We also found that this aromatic slot comprised a unique topology, not only for histamine receptors, but also for other aminergic receptors (Supplementary Fig. 6). The fact that residues in positions 7.39 and 7.42 of H_4_R were less conserved in other receptors supports that this sub-pocket in H_4_R is highly distinctive, and targeting this aromatic slot in H_4_R may be useful for the design of compounds specific to H_4_R (or H_3_R). Although the isothiourea group of imetit did not appear to be a substituent developed for the aromatic slot, our structure indicated that it was a substitution that could effectively exploit the slot.

As mentioned in the previous section, aromatic clusters on TM6 (Trp^6.48^, Phe/Tyr^6.51^, and Phe^6.52^) play an important role in the binding of agonists and antagonists at aminergic receptors^25,28^. In H_1_R_his_, the imidazole ring of histamine also interacted with this aromatic cluster and formed a hydrophobic interaction with Phe435^6.55^ within the Van Der Waals radius (Supplementary Fig. 7c). However, in H_4_R, some of the residues constituting this aromatic cluster were compact polar residues (Ser320^6.52^ and Thr323^6.55^) and created a wider polar subpocket between TM5 and 6 (Supplementary Fig. 7a, b). Since the residues in this position were distinguishable between H_1_R/H_2_R and H_3_R/H_4_R, the effective utilization of this pocket with aromatic substituents may have been important for generating selectivity in histamine receptor subtypes (Fig. 4d).

### Activation mechanism of H_4_R

**Fig. 5:**
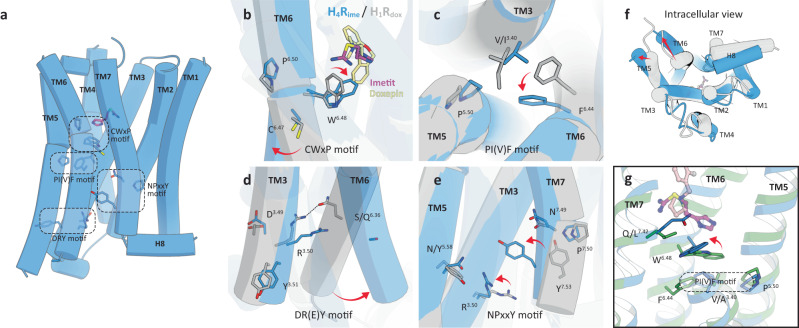
Activation mechanism of H_4_R. **a** Activation motifs in the H_4_R structure. Each motif residue is shown as a blue stick. **b**–**g** Structural comparison between the active H_4_R_ime_ (blue) and antagonist-bound inactive H_1_R_his_ (gray, PDB 3RZE). (**b**) CwxP motif, (**c**) PI(V)F motif, (**d**) DR(E)Y motif, (**e**) NPxxY motif. Imetit and doxepin are shown as magenta and khaki sticks. The residues involved in receptor activation are indicated by sticks, and conformational changes are shown as red arrows. Microswitch rearrangement allowed movement outside the cytoplasmic ends of TM5 and 6 (**f**). **g** Gln347^7.42^ coordination and its effect on surrounding residues.

Since the inactive structure of H_4_R has not yet been elucidated, we assessed the activation mechanism of H_4_R by comparing the inactive structure of antagonist-bound H_1_R (H_1_R_dox_)^19^ with our agonist-bound H_4_R structure H_4_R_ime_. Four motif structures (CWxP, PI(V)F, DR(E)Y, and NPxxY) that are important for GPCR activation are conserved in H_4_R; therefore, it is valid to compare H_4_R_ime_ and inactive H_1_R_dox_ to assess these motif structures and the activation mechanism of H_4_R (Fig. 5a). Different pharmacological features exist for each ligand that binds to the GPCR. This is thought to be the result of the orientation of the side chains of this conserved local microswitch that affects the global movement of the helical backbone^37,38^. In agonist-bound H_4_R_ime_, a hydrophobic interaction between the agonist and Trp316^6.48^ (known as the “toggle switch”) of TM6 was observed and caused a downward swing in the side chain (Fig. 5b). This conformational change in Trp316^6.48^ promoted the rearrangement of the unique configuration of Pro186^5.50^-Val102^3.40^-Phe312^6.44^ (PIF motif in other GPCRs) and Asp111^3.49^-Arg112^3.50^-Tyr113^3.51^ (DR(E)Y motif), thus allowing movement outside the cytoplasmic end of TM6 (Fig. 5c, d, f). The NPxxY motif at the cytoplasmic end of TM7 also showed a conformational change in the side chain rotamer of Tyr358^7.53^. This conformational change moved TM7 toward the TM3 and 5 sides, a distance that allowed an interaction with the DR(E)Y motif (Fig. 5e). Thus, the conformational changes of the microswitch and global movement of the TM bundle, which are commonly observed in GPCRs, were conserved in our structure. This confirmed that our H_4_R_his_ and H_4_R_ime_ structures are the active conformations that interact with trimeric G proteins.

Recently, the inactive structures of the antagonist-bound histamine H_2_ receptor (H_2_R) and the histamine H_3_ receptor (H_3_R) were published^39,40^, and comparison of our agonist-bound H_4_R structure with the PF-03654746-bound inactive H_3_R structure suggests that the activation mechanism in H_3_R/H_4_R is different from that in H_1_R. As mentioned above, in the case of many aminergic receptors, the agonist interacts with the toggle switch, causing a downward swing of its side chain and rearrangement of the downstream structure to the active form. In the case of H_3_R/H_4_R, however, the present structure shows that the side chain of the toggle switch is not moved perpendicularly but horizontally (Fig. 5g). In H_4_R, the agonist formed the hydrophobic interaction with Trp316^6.48^ and changes its side chain about 90° to the TM3 side, which may serve as a trigger in receptor activation. Here, the residue at position 7.42 may play an important role in controlling the conformational change of the toggle switch horizontally. The side chain of Gln347^7.42^ in the cryo-EM structure of H_4_R is extended to the TM6 side, which is consistent with Leu401^7.42^ conformation in the H_3_R structure. In most aminergic receptors, position 7.42 is conserved in Gly (only muscarinic acetylcholine receptors have Cys), but only H_3_R/H_4_R have Leu/Gln, which have larger side chains than Gly (Supplementary Fig. 8). In other words, the toggle switch within the H_3_R/H_4_R appears to have a unique movement, characterized by a lateral movement that maintains the “knock over” state to prevent steric hindrance with Leu/Gln^7.42^.

## Discussion

In this study, we present the cryo-EM structures of the histamine H4 receptor bound by two agonists histamine and imetit, respectively, which exhibit distinct pharmacological properties. Although G_q_ signal activity could not be measured directly in the in-house measurement, weak G_q_ coupling was reported in the previous study^21^. The tethering system used in the present study effectively brings GPCRs and trimeric G proteins into close proximity, which is thought to make the binding with G_q_ stronger, enabling structural analysis.

In histamine receptor subtypes, H_4_R has low sequence similarities with H_1_R and H_2_R but has a more similar sequence identity with H_3_R. Given the overlap of the sequences, it would be difficult to develop a ligand that is H_4_R-selective and H_3_R-nonselective. Since the active structure of H_3_R is currently unknown, we generated a homology model of active state H_3_R based on the H_4_R structure revealed in the present study and compared their ligand recognition sites (Supplementary Fig. 9). Although the orthosteric binding sites in both receptors were expected to be similar, there were differences in some of the residues involved in ligand recognition (Fig. 4d, Supplementary Figs. 9a–c). Histamine had almost the same affinity for H_3_R/H_4_R, but OUP-16, which is an imidazole derivative agonist, exhibited 16-fold selectivity for H_4_R (H_3_R K_i_ = 2 μM / H_4_R K_i_ = 125 nM) (Supplementary Fig. 10)^15^. To investigate the difference in selectivity, we also performed a docking simulation of OUP-16 for the cryo-EM structure of H_4_R obtained in the present study (Supplementary Fig. 9d). Comparing the docking result of OUP-16 and homology model of H_3_R may explain the differences in their affinity. The docking simulation indicated that the guanidine moiety of OUP-16 engages in hydrogen bonding with Asp94. The imidazole ring on the opposite side of the agonist exhibits an alternative binding mode compared to the imidazole ring of histamine and is oriented toward the extracellular ends of TM5 and 6. Furthermore, the hydrogen bond with Glu182 is maintained and the interaction with Ser320 is established. The entire imidazole ring is occupied within the sub-pocket formed between TM4, 5, and 6. Within this pocket, however, there were different residues (Y/N^4.57^, T/S^6.52^, M/T^6.55^) for H_3_R/H_4_R, which may have resulted in the differences in pocket shape and properties in the two receptors (Supplementary Fig. 9a–c, d). The coordination of the hydrophobic and massive residues Tyr167^4.57^ and Met378^6.55^ next to Glu206^5.46^, which is essential for hydrogen bonding with histamine in H_3_R, implies that the introduction of an additional sub-pocket is not tolerated. Therefore, the design of ligands that take into account the shape of the orthosteric binding pocket specific to H_3_R and H_4_R would allow for their respective selectivity to be generated. JNJ-7777120 was the first H_4_R-selective antagonist to be discovered and has contributed significantly to our understanding of the pathophysiological functions of H_4_R (Supplementary Fig. 10)^41^. In addition, the compound has interesting pharmacological characteristics, such as biased activity toward β-arrestin^42,43^. Several antagonists derived from this compound have been demonstrated to be effective against pruritus, dermatitis, asthma, and arthritis in preclinical studies in animal models. Clinical trials have been conducted in some cases^44,45^. Since ligands that achieve such pharmacological effects require a high selectivity for H_4_R, the structures of H_4_R revealed in the present study may provide information that could facilitate the development of H_4_R-selective ligands. We also performed a docking study on JNJ-7777120 (Supplementary Fig. 9e). The results show that the H_4_R-specific sub-pocket provides space to accept the chloroindole ring of JNJ-7777120, which is not present in other histamine receptors, including H_3_R, and is expected to be an important region that allows subtype selectivity to be acquired. In addition, mutant experiments in previous reports have demonstrated that Asn147^4.57^, Phe169^ECL2^, Leu175^5.39^, and Ser179^5.43^, which correspond to this sub-pocket, decrease the activity of JNJ-777120, which could support our structural considerations^31,46^.

Acquiring the inactive structure of H_4_R is a future challenge that may yield a better understanding of the antagonist binding and the activation mechanism of H_4_R. The structural, biological, and pharmacological results of the H_4_R obtained in this study provide details on the molecular mechanisms of agonism in H_4_R and key insights into the mechanism of selectivity in histamine receptor subtypes. A rational structure-based design for effective drugs against CIDs may be developed further using the structural information on H_4_R presented herein.

## Methods

### Constructs

The full-length human H_4_R (residues 1–390) was subcloned into a pFastBac1 plasmid with an N-terminal hemagglutinin (HA) signal peptide, a FLAG tag, and 8× His tag, followed by a thermostabilized apocytochrome b_562_RIL (bRIL) epitope^47^ to facilitate protein expression and stability (Supplementary Fig. 1a). An HRV 3 C protease cleavage site and linker residues were inserted between the H_4_R and bRIL. The artificial G_q_ used in the present study was based on the mini-G_s_ skeleton, which has two dominant-negative mutations and the alpha-helical domain truncation^48^. The 35 amino acids of the N-terminal were replaced with the corresponding sequence of Gα_i1_ to facilitate scFv16 binding^49,50^. Such an approach was used to obtain the structures of G_q_-bound 5-HT_2A_ receptor/ghrelin receptor and G_11_-bound M_1_ receptors^51–53^. Furthermore, G_i_ (DNG_i1_) corresponding to Supplementary Fig. 1b was generated by introducing four dominant-negative mutations, S47N, G203A, A326S, and E245A^50^. To improve the stability and homogeneity of the receptor-G protein complex, we employed the NanoBiT tethering system with a large fragment of NanoLuc (LgBiT) and high affinity 11 amino acid peptide (HiBiT) tag attached to the C-terminus of the receptor and Gβ_1_, respectively^54^. G_q_, Nb35, scFv16, and Ric8A were cloned into the pFastBac1 plasmid. His_10_-rat Gβ_1_ with C-terminal HiBiT and bovine Gγ_2_ were subcloned into the pFastBac Dual vector.

### Protein expression and complex purification

Recombinant baculoviruses were prepared using the Bac-to-Bac baculovirus expression system (Invitrogen, Carlsbad, CA). *Spodoptera frugiperda* (Sf9) insect cells were grown to a density of 2–3 × 10^6^ cells/mL and co-infected with H_4_R, G_q_, Gβ_1_Gγ_2_, Nb35, scFv16, and Ric8A viral stocks at a multiplicity of infection ratio of 8:2:2:1:1:1. They were harvested 48 h later. Cell pellets were resuspended in a buffer containing 20 mM of HEPES (pH 7.5), 100 mM of NaCl, 10% glycerol, 5 mM of MgCl_2_, 5 mM of CaCl_2_, 0.25 mM of TCEP, protease inhibitor cocktail (Nacalai Tesque), 25 mU/mL of apyrase, and 50 μM of H_4_R agonists (histamine or imetit) followed by incubation for 1 h at room temperature. Cell membranes were collected by centrifugation and solubilized in Solubilization Buffer containing 20 mM of HEPES (pH 7.5), 100 mM of NaCl, 10% glycerol, 5 mM of MgCl_2_, 5 mM of CaCl_2_, 0.25 mM of TCEP, protease inhibitor cocktail, 25 mU/mL of apyrase, 0.5% (w/v) lauryl maltose neopentyl glycol (LMNG, Anatrace) with 0.05% (w/v) cholesteryl hemisuccinate (CHS, Sigma-Aldrich), and 50 μM of agonists, for 2 h at 4 °C.

Insoluble materials were removed by centrifugation, and the supernatants were incubated with TALON metal affinity resin (Clontech) overnight at 4 °C. The resin was washed with 10 column volumes (CV) of Wash Buffer containing 20 mM of HEPES (pH 7.5), 100 mM of NaCl, 10% glycerol, 2 mM of MgCl_2_, 2 mM of CaCl_2_, 20 mM of imidazole, 0.25 mM of TCEP, 0.01% (w/v) LMNG, 0.01% (w/v) GDN (Anatrace), 0.001% (w/v) CHS, and 20 μM of agonists. The complex was then eluted with 3 CV of Elution Buffer containing 20 mM of HEPES (pH 7.5), 100 mM of NaCl, 10% glycerol, 2 mM of MgCl_2_, 2 mM of CaCl_2_, 250 mM of imidazole, 0.25 mM of TCEP, 0.01% (w/v) LMNG, 0.001% (w/v) GDN (Anatrace), 0.001% (w/v) CHS, and 20 μM of agonists. The N-terminal FLAG tag and bRIL epitope were cleaved by an HRV 3 C protease for 3 h at 4 °C. Finally, the complex was concentrated to 0.5 mL using the Amicon Ultra-15 concentrator (Millipore) and subjected to size-exclusion chromatography on a Superdex 200 Increases 10/300 column (GE Healthcare) pre-equilibrated with a buffer containing 20 mM of HEPES (pH 7.5), 100 mM of NaCl, 2 mM of MgCl_2_, 2 mM of CaCl_2_, 0.25 mM of TCEP, 0.0015% (w/v) LMNG, 0.0005 (w/v) GDN, 0.00015% (w/v) CHS, and 20 μM of agonists. The fractions containing the monomeric complex were collected and concentrated to approximately 10 mg/mL for electron microscopy experiments. Expression and purification of the H_4_R-G_i_ complex (corresponding to Supplementary Fig. 1b) was performed similar to that for G_q_ above. However, since Nb35 does not bind to G_i_, Nb35 co-expression was not performed.

### Cryo-EM grid preparation and data collection

The Quantifoil R1.2/1.3 holy carbon copper grid (Quantifoil) was glow-discharged at 7 Pa with 10 mA for 10 s using a JEC-3000FC sputter coater (JEOL) prior to use. A 3-μL aliquot was applied to the grid, blotted for 3.5 s with a blot force of 10 in 100% humidity at 8 °C, and plunged into liquid ethane using a Vitrobot Mark IV (Thermo Fisher Scientific). Cryo-EM data collection for screening sample quality and grid conditions was performed using a Glacios Cryo-transmission electron microscope operated at 200 kV accelerating voltage with a Falcon4 camera (Thermo Fisher Scientific) at the Institute for Life and Medical Sciences, Kyoto University. After several screening sessions, data were collected using a Titan Krios (Thermo Fisher Scientific) at 300 kV accelerating voltage equipped with a direct K3 electron detector, Gatan BioQuantum energy filter (slit width of 20 eV) (Gatan), and Cs corrector (CEOS, GmbH), which were installed at the Institute for Protein Research, Osaka University. Data collection was carried out using SerialEM software^55^ at a norminal magnification of ×81,000 (calibrated pixel size of 0.88 Å pixel^−1^) with a total exposure time of 5.0 s (50 frames) with a defocus range of –0.8 to –2.0 μm. The detailed imaging conditions are described in Table 1.

### Cryo-EM data processing

All image processing was performed using cryoSPARC^56^. We manually inspected and curated the micrographs after patch-based motion correction and contrast transfer function (CTF) estimation. The particles for the histamine-bound-H_4_R-G_q_ complex were selected using the Blob particle picker and initial two-dimensional (2D) classification yielded templates for subsequent template picking. A subset of the selected particles was used as a training set for Topaz, which was used to repick the particles from the micrographs^57^. The particles for the imetit-bound H_4_R-G_q_ complex were picked using the automated procedure in crYOLO^58^. The picked particles were subjected to a 2D classification to discard particles in poorly defined classes. Ab initio reconstruction was performed in cryoSPARC asking for four classes, which resulted in one good class and three trash classes. Multiple rounds of heterogeneous refinement were then performed against the four ab initio models to remove bad particles. The selected particles were extracted at full pixel size and subjected to non-uniform (NU) refinement^59^. Further classification without alignment was performed using a soft protein mask to create a better-looking ligand binding region for the histamine-bound-H_4_R-G_q_ complex.

The densities were further Improved by per-particle defocus refinement and another round of non-uniform refinement to generate the final map. Resolutions were estimated using the ‘gold standard’ criterion (FSC = 0.143). The local resolution was calculated in cryoSPARC. Map sharpening was reevaluated with the Phenix autosharpen tool^60,61^. These maps were used for modeling. The validation on the coordinate refinement (FSC_work_/FSC_test_) was calculated using servalcat^62^. The processing strategy is described in Supplementary Fig. 2.

### Model building and refinement

Model building was facilitated by both the previous cryo-EM structures of the GHSR-G_q_ complex (PDB 7F9Z) and H_1_R bound to histamine (PDB 7DFL)^20,53^. The receptor, G_q_ trimer, Nb35, and scFv16 models were manually built in Coot^63^, followed by several rounds of real-space refinement using Phenix^64^. All molecular graphics were prepared using CueMol (http://www.cuemol.org) and UCSF ChimeraX^65^. The 3D reconstruction and model refinement statistics are summarized in Table 1.

### Molecular docking

The docking study was performed using H_4_R_ime_ with the Glide program^66^ in the Schrödinger Suite 2022-4 (Schrödinger LLC). The coordinates of OUP-16 and JNJ-7777120 were initially built using LigPrep in the Schrödinger Suite at pH 7.0 and the OPLS3 force field. Each ligand was docked into the binding pocket of H_4_R, resulting in ten conformations per ligand. The most reliable binding poses were selected based on the interaction energy and visual inspection.

### TGFα shedding assay

The mutants were prepared using the primers listed in Supplementary Table 2. The activity of the endogenous agonist (histamine) and its derivative (imetit) for H_4_R mutants on G protein signaling was determined using a TGFα shedding assay. In brief, a pCAGGS plasmid encoding the human wild-type or a mutant H_4_R (human, full-length, and untagged), together with pCAGGS plasmids that encoded the chimeric Gα_q/i1_ subunit and alkaline phosphatase-tagged TGFα (AP-TGFα; human codon-optimized), were transfected into HEK293A cells by a Lipofectamine® 3000 (LFA) transfection reagent (Thermo Fisher Scientific) (125 ng of the H_4_R plasmid, 125 ng of the Gα_q/i1_ plasmid, 625 ng of the AP-TGFα plasmid, and 18.2 μL of LFA per well in a six-well culture plate). The chimeric Gα_q/i1_ subunit comprises the Gα_q_ backbone and G_αi1_-derived six-amino acid C-terminus and couples with G_i_-coupled H_4_R but induces a G_q_-dependent TGFα shedding response^24^. After culturing for 1 day and incubating at 37 °C in a 5% CO_2_ incubator, the transfected cells were harvested by trypsinization, neutralized with Dulbecco’s modified Eagle’s medium containing 10% fetal calf serum (FCS) and penicillin–streptomycin, washed once with Hank’s balanced salt solution (HBSS) containing 5 mM of HEPES (pH 7.4), and resuspended in 6 mL of the HEPES-containing HBSS. The cell suspension was seeded into a 96-well plate at a volume of 90 mL per well (typically, 48 wells per transfected cells) and incubated for 30 min in the CO_2_ incubator. Test compounds (10×, diluted in 0.01% BSA and 5 mM of HEPES-containing HBSS, 10 mL volume) were added to duplicate wells and incubated for 1 h. After centrifugation, the conditioned medium (80 mL) was transferred to an empty 96-well plate. AP reaction solution [10 mM of *p*-nitrophenylphosphate (*p*-NPP), 120 mM of Tris–HCl (pH 9.5), 40 mM of NaCl, and 10 mM of MgCl_2_] was dispensed into the cell culture plates and plates containing conditioned media (80 mL). Absorbance was measured at 405 nm before and after a 1-h incubation period at 25 °C using a microplate reader (iMark™ Microplate Absorbance Reader; BioRad). Ligand-induced AP-TGFα release was calculated as previously described^24^. The vehicle-treated AP-TGFα release signal was set as a baseline unless otherwise specified. AP-TGFα release signals were fitted with a four-parameter sigmoidal concentration-response curve, from which EC_50_ and E_max_ values were obtained, using GraphPad Prism 9 software (GraphPad Prism). Negative values of logarithmically transformed EC_50_ values (pEC_50_) were used to calculate the mean and standard error of independent experiments.

To estimate the expression level of each H_4_R mutant, a FLAG tag was added to the N-terminus of all mutants. Transfection of the genes into the cells was performed as described above, and the FLAG-tagged mutant genes were transfected at the same time as all the genes required for the TGFα shedding assay. One day after gene transfer, transfected cells were harvested and stained with anti-DYKDDDDK tag monoclonal antibody (FUJIFILM Wako Pure Chemical Corporation) as primary antibody and Goat anti-mouse IgG antibody conjugated with Alexa Fluor™ 488 (Thermo Fisher Scientific) as a secondary antibody for 30 min on ice in the dark. Stained cells were replaced with FACS buffer (PBS(-) with 2% FCS and 0.1% NaN_3_), and cells were assayed using a flow cytometer (Guava EasyCyte Plus, Millipore). The expression levels of the mutant were estimated in terms of the mean fluorescence intensity (MFI) ratio (MFI of the mutant compared to that of the WT (Supplementary Fig. 4a).

### Statistical analysis

All functional study data were analyzed using Prism 9 (GraphPad) and are presented as mean ± standard error of the mean (SEM) of three independent experiments. Statistical analyses were performed using Prism 9 (GraphPad) with one-way analysis of variance followed by Dunnett’s post-hoc test. Values with *p* < 0.05 are considered statistically significant.

### Reporting summary

Further information on research design is available in the Nature Portfolio Reporting Summary linked to this article.

### Supplementary information

Supplementary Information

Peer Review File

Reporting Summary

### Source data

Source Data

## Data Availability

The cryo-EM density maps and atomic coordinates have been deposited in the Electron Microscopy Data Bank (EMDB) and wwPDB under accession numbers EMD-33785 and 7YFC [10.2210/pdb7YFC/pdb] for the histamine-bound H_4_R-G_q_ complex, EMD-33786 and 7YFD [10.2210/pdb7YFD/pdb] for the imetit-bound H_4_R-G_q_ complex. Raw movies were deposited to EMPIAR data base (https://www.ebi.ac.uk/empiar/) with accession numbers EMPIAR-11708. Source data are provided with this paper.

## References

[1] Panula P (2015). International union of basic and clinical pharmacology. XCVIII. histamine receptors. Pharmacol. Rev..

[2] Ash ASF, Schild HO (1966). Receptors mediating some actions of histamine. Br. J. Pharmacol. Chemother..

[3] Black JW, Duncan WAM, Durant CJ, Ganellin CR, Parsons EM (1972). Definition and antagonism of histamine H2-Receptors. Nature.

[4] Thurmond RL (2015). The histamine H4 receptor: From orphan to the clinic. Front. Pharmacol..

[5] Arrang JM, Garbarg M, Schwartz JC (1983). Auto-inhibition of brain histamine release mediated by a novel class (H3) of histamine receptor. Nature.

[6] Lovenberg TW (1999). Cloning and functional expression of the human histamine H3 receptor. Mol. Pharmacol..

[7] Schwartz JC (2011). The histamine H3 receptor: From discovery to clinical trials with pitolisant. Br. J. Pharmacol..

[8] Oda T, Morikawa N, Saito Y, Masuho Y, Matsumoto SI (2000). Molecular cloning and characterization of a novel type of histamine receptor preferentially expressed in leukocytes. J. Biol. Chem..

[9] Nakamura T, Itadani H, Hidaka Y, Ohta M, Tanaka K (2000). Molecular cloning and characterization of a new human histamine receptor, HH4R. Biochem. Biophys. Res. Commun..

[10] Liu C (2001). Cloning and pharmacological characterization of a fourth histamine receptor (H4) expressed in bone marrow. Mol. Pharmacol..

[11] Morse KL (2001). Cloning and characterization of a novel human histamine receptor. J. Pharmacol. Exp. Ther..

[12] Nguyen T (2001). Discovery of a novel member of the histamine receptor family. Mol. Pharmacol..

[13] Gutzmer R (2011). Pathogenetic and therapeutic implications of the histamine H4 receptor in inflammatory skin diseases and pruritus. Front. Biosci..

[14] Cramp S (2010). Identification and hit-to-lead exploration of a novel series of histamine H4 receptor inverse agonists. Bioorganic Med. Chem. Lett..

[15] Marson CM (2011). Targeting the histamine H4 receptor. Chem. Rev..

[16] Zampeli E, Tiligada E (2009). The role of histamine H 4 receptor in immune and inflammatory disorders. British J. Pharmacol..

[17] Sadek B, Stark H (2016). Cherry-picked ligands at histamine receptor subtypes. Neuropharmacology.

[18] Corrêa MF, Fernandes JPDS (2015). Histamine H4 receptor ligands: Future applications and state of art. Chem. Biol. Drug Des..

[19] Shimamura T (2011). Structure of the human histamine H 1 receptor complex with doxepin. Nature.

[20] Xia R (2021). Cryo-EM structure of the human histamine H1 receptor/Gq complex. Nat. Commun..

[21] Inoue A (2019). Illuminating G-protein-coupling selectivity of GPCRs. Cell.

[22] Rasmussen SGF (2011). Crystal structure of the β2 adrenergic receptor–Gs protein complex. Nat. 2011 4777366.

[23] Maeda, S. et al. Development of an antibody fragment that stabilizes GPCR/G-protein complexes. *Nat. Commun*. **9**, (2018). 10.1038/s41467-018-06002-w PMC6137068 30213947

[24] Inoue A (2012). TGFα shedding assay: An accurate and versatile method for detecting GPCR activation. Nat. Methods.

[25] Michino M (2015). What can crystal structures of aminergic receptors tell us about designing subtype-selective ligands?. Pharmacol. Rev..

[26] Zhuang Y (2021). Structural insights into the human D1 and D2 dopamine receptor signaling complexes. Cell.

[27] Im D (2020). Structure of the dopamine D2 receptor in complex with the antipsychotic drug spiperone. Nat. Commun..

[28] Kooistra AJ, Kuhne S, De Esch IJP, Leurs R, De Graaf C (2013). A structural chemogenomics analysis of aminergic GPCRs: Lessons for histamine receptor ligand design. Br. J. Pharmacol..

[29] Jongejan A (2008). Delineation of agonist binding to the human histamine H4 receptor using mutational analysis, homology modeling, and ab initio calculations. J. Chem. Inf. Model..

[30] Shin N (2002). Molecular modeling and site-specific mutagenesis of the histamine-binding site of the histamine H4 receptor. Mol. Pharmacol..

[31] Lim HD (2010). Molecular determinants of ligand binding to H4R species variants. Mol. Pharmacol..

[32] van der Goot H, Schepers M, Sterk G, Timmerman H (1992). Isothiourea analogues of histamine as potent agonists or antagonists of the histamine H3-receptor. Eur. J. Med. Chem..

[33] Garbarg M (1992). S-[2-(4-imidazolyl)ethyl]isothiourea, a highly specific and potent histamine H3 receptor agonist.. J. Pharmacol. Exp. Ther..

[34] Mehta, P. et al. Enigmatic histamine receptor h4 for potential treatment of multiple inflammatory, autoimmune, and related diseases. *Life* **10**, (2020). 10.3390/life10040050 PMC7235846 32344736

[35] Feng Z, Hou T, Li Y (2013). Docking and MD study of histamine H4R based on the crystal structure of H1R. J. Mol. Graph. Model..

[36] Schultes S (2013). Mapping histamine H4 receptor-ligand binding modes. Medchemcomm.

[37] Katritch V, Cherezov V, Stevens RC (2013). Structure-function of the G protein-coupled receptor superfamily. Annu. Rev. Pharmacol. Toxicol..

[38] Filipek S (2019). Molecular switches in GPCRs. Curr. Opin. Struct. Biol..

[39] Robertson MJ (2022). Structure determination of inactive-state GPCRs with a universal nanobody. Nat. Struct. Mol. Biol..

[40] Peng, X. et al. Structural basis for recognition of antihistamine drug by human histamine receptor. *Nat. Commun*. **13**, (2022). 10.1038/s41467-022-33880-y PMC9569329 36243875

[41] Jablonowski JA (2003). The first potent and selective non-imidazole human histamine H4 receptor antagonists. J. Med. Chem..

[42] Rosethorne EM, Charlton SJ (2010). Agonist-biased signalling at the histamine H4 receptor: JNJ7777120 recruits beta-arrestin without activating G proteins. Mol. Pharmacol..

[43] Nijmeijer S (2013). Detailed analysis of biased histamine H4 receptor signalling by JNJ 7777120 analogues. Br. J. Pharmacol..

[44] Thurmond RL (2014). Clinical and preclinical characterization of the histamine H(4) receptor antagonist JNJ-39758979. J. Pharmacol. Exp. Ther..

[45] Murata Y (2015). Phase 2a, randomized, double-blind, placebo-controlled, multicenter, parallel-group study of a H4 R-antagonist (JNJ-39758979) in Japanese adults with moderate atopic dermatitis. J. Dermatol..

[46] Wifling D (2015). Molecular determinants for the high constitutive activity of the human histamine H4 receptor: Functional studies on orthologues and mutants. Br. J. Pharmacol..

[47] Chun E (2012). Fusion partner toolchest for the stabilization and crystallization of G protein-coupled receptors. Structure.

[48] Nehmea R (2017). Mini-G proteins: Novel tools for studying GPCRs in their active conformation. PLoS One.

[49] Carpenter B, Nehmé R, Warne T, Leslie AGW, Tate CG (2016). Structure of the adenosine A2A receptor bound to an engineered G protein. Nature.

[50] Liang YL (2018). Dominant negative g proteins enhance formation and purification of agonist-gpcr-g protein complexes for structure determination. ACS Pharmacol. Transl. Sci..

[51] Kim K (2020). Structure of a hallucinogen-activated Gq-coupled 5-HT2A serotonin receptor. Cell.

[52] Maeda S, Qu Q, Robertson MJ, Skiniotis G, Kobilka BK (2019). Structures of the M1 and M2 muscarinic acetylcholine receptor/G-protein complexes. Science (80-.)..

[53] Wang Y (2021). Molecular recognition of an acyl-peptide hormone and activation of ghrelin receptor. Nat. Commun..

[54] Duan J (2020). Cryo-EM structure of an activated VIP1 receptor-G protein complex revealed by a NanoBiT tethering strategy. Nat. Commun..

[55] Mastronarde DN (2005). Automated electron microscope tomography using robust prediction of specimen movements. J. Struct. Biol..

[56] Punjani A, Rubinstein JL, Fleet DJ, Brubaker MA (2017). CryoSPARC: Algorithms for rapid unsupervised cryo-EM structure determination. Nat. Methods.

[57] Bepler T, Kelley K, Noble AJ, Berger B (2020). Topaz-Denoise: general deep denoising models for cryoEM and cryoET. Nat. Commun..

[58] Wagner T (2019). SPHIRE-crYOLO is a fast and accurate fully automated particle picker for cryo-EM. Commun. Biol..

[59] Punjani A, Zhang H, Fleet DJ (2020). Non-uniform refinement: adaptive regularization improves single-particle cryo-EM reconstruction. Nat. Methods.

[60] Terwilliger TC, Sobolev OV, Afonine PV, Adams PD (2018). Automated map sharpening by maximization of detail and connectivity. Acta Crystallogr. Sect. D Struct. Biol..

[61] Adams PD (2010). PHENIX: A comprehensive Python-based system for macromolecular structure solution. Acta Crystallogr. Sect. D Biol. Crystallogr..

[62] Yamashita K, Palmer CM, Burnley T, Murshudov GN (2021). Cryo-EM single-particle structure refinement and map calculation using Servalcat. Acta Crystallogr. Sect. D Struct. Biol..

[63] Emsley P, Lohkamp B, Scott WG, Cowtan K (2010). Features and development of Coot. Acta Crystallogr. Sect. D Biol. Crystallogr..

[64] Afonine PV (2018). Real-space refinement in PHENIX for cryo-EM and crystallography. Acta Crystallogr. Sect. D Struct. Biol..

[65] Goddard TD (2018). UCSF ChimeraX: Meeting modern challenges in visualization and analysis. Protein Sci..

[66] Friesner RA (2004). Glide: A New Approach for Rapid, Accurate Docking and Scoring. 1. Method and Assessment of Docking Accuracy. J. Med. Chem..

