# Supplementary material for: Structural insights into the agonists binding and receptor selectivity of human histamine H4 receptor
Source: Nat Commun. 2023 Oct 20;14:6538. doi: 10.1038/s41467-023-42260-z (PMC10589313; doi:10.1038/s41467-023-42260-z)
Supplement: Supplementary file 1 — Supplementary Information [file 41467_2023_42260_MOESM1_ESM.pdf]

Supplementary Information

Structural Insights into the Agonists Binding and Receptor Selectivity of Human Histamine H_4 Receptor

D. Im *et al.*

a

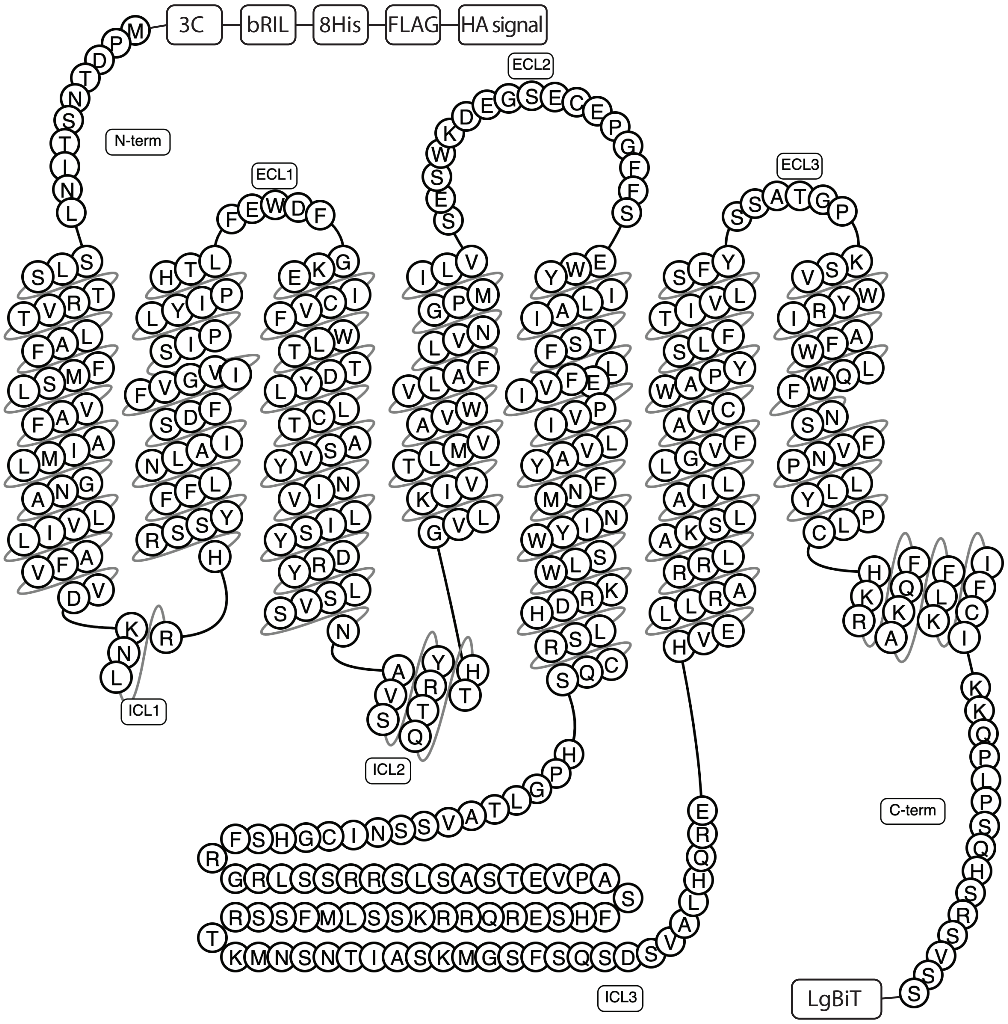

b

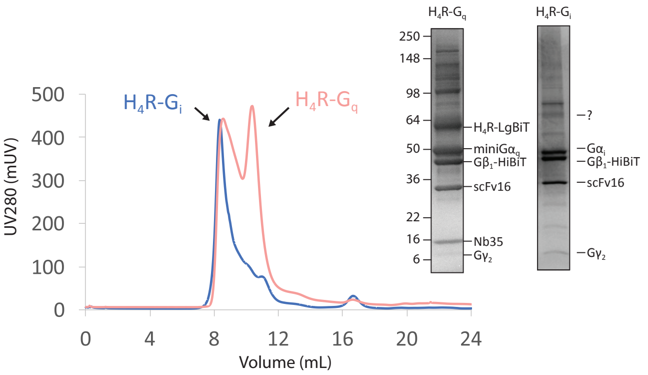

Supplementary Figure 1 | Sample preparation of H_4R-G_q complex.

a, Snake-shape diagram of the H_4R-LgBiT construct used in complex assembling. The snake-diagram of H_4R was adopted and modified from the GPCRdb (https://www.gpcrdb.org). **b**, Size exclusion chromatography profiles of H_4R/G_q and H_4R/G_i complexes.

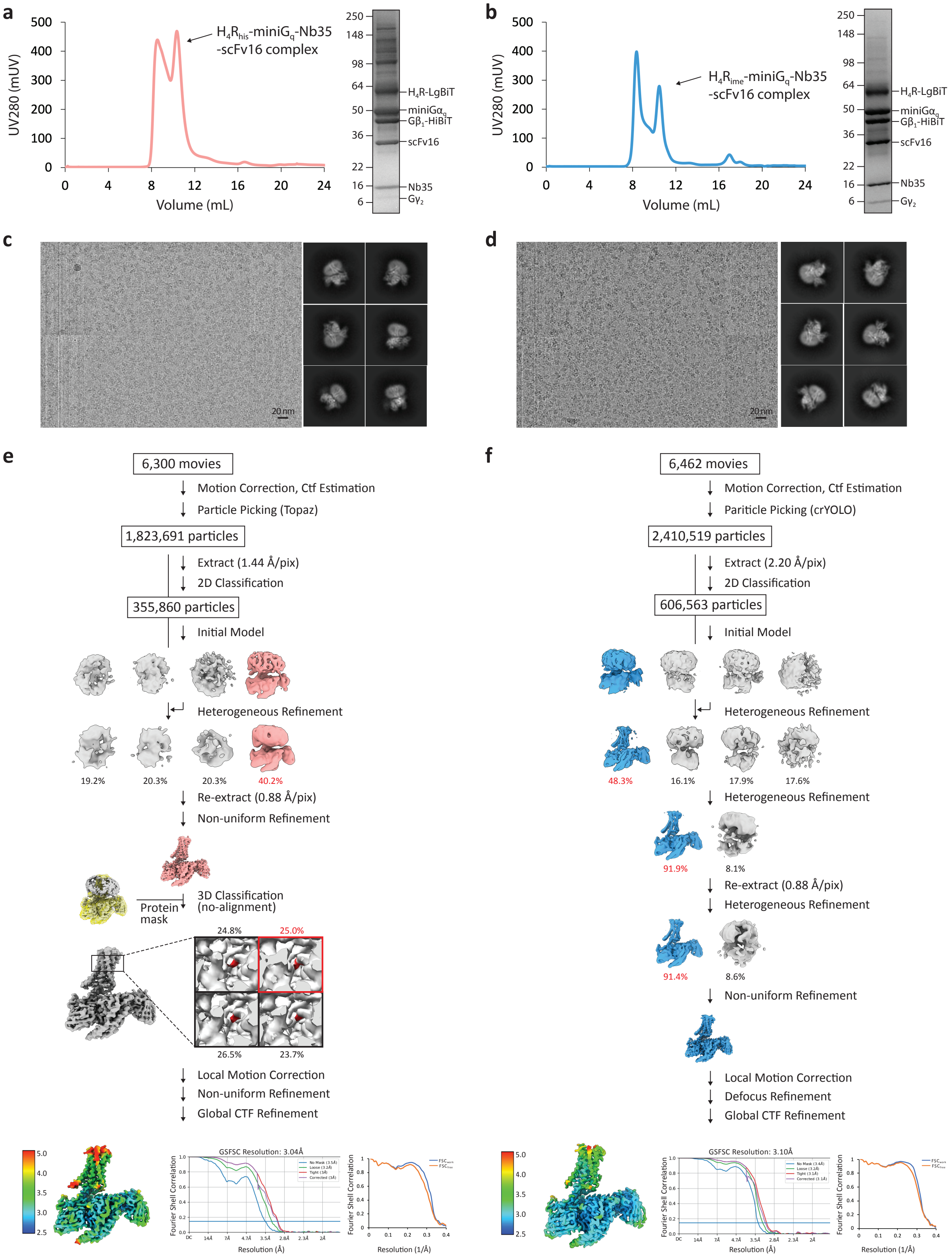

Supplementary Figure 2 | Single-particle cryo-EM analysis procedure.

a, **b**, Size exclusion chromatography profiles and SDS-PAGE analysis of the histamine-bound H_4R-G_q (**a**) and imetit-bound H_4R-G_q complexes (**b**). **c**, **d**, Representative cryo-EM micrographs and two-dimensional classification averages of the histamine-bound H_4R-G_q (**c**) and imetit-bound H_4R-G_q complexes (**d**). **e**, **f**, Cryo-EM data processing workflows of the histamine-bound H_4R-G_q (**e**) and imetit-bound H_4R-G_q complexes (**f**).

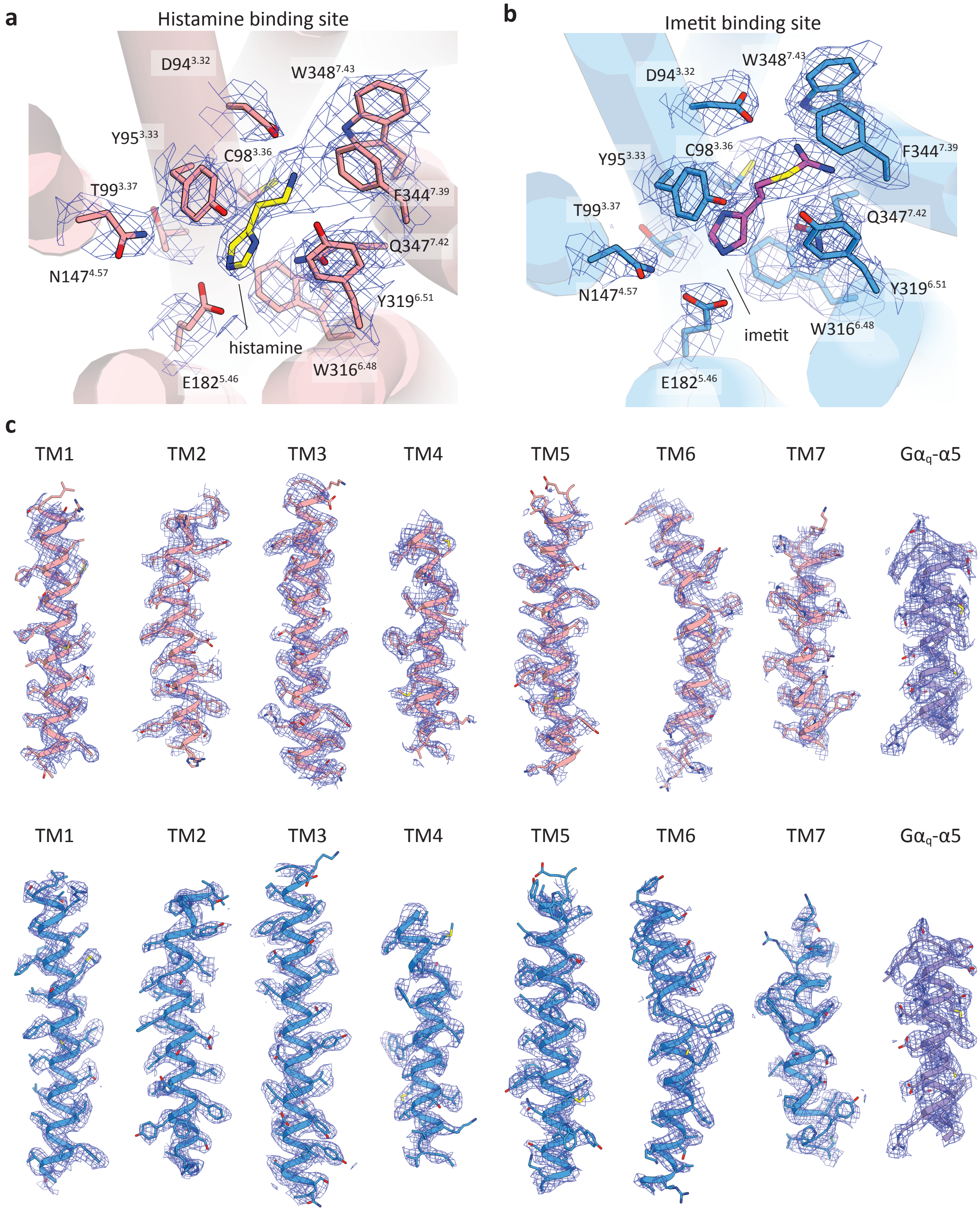

Supplementary Figure 3 | Cryo-EM density map.

a, **b**, The agonist-binding site of the histamine-bound H$_4$R-G$_q$ (**a**) and imetit-bound H$_4$R-G$_q$ complexes (**b**). **c**, The transmembrane helices 1–7, helix 8, and the α5 helix of the G$_q$ of the histamine-bound H$_4$R-G$_q$ (top panel) and imetit-bound H$_4$R-G$_q$ complexes (lower panel). The density map was drawn by CueMol at contour level 3.0.

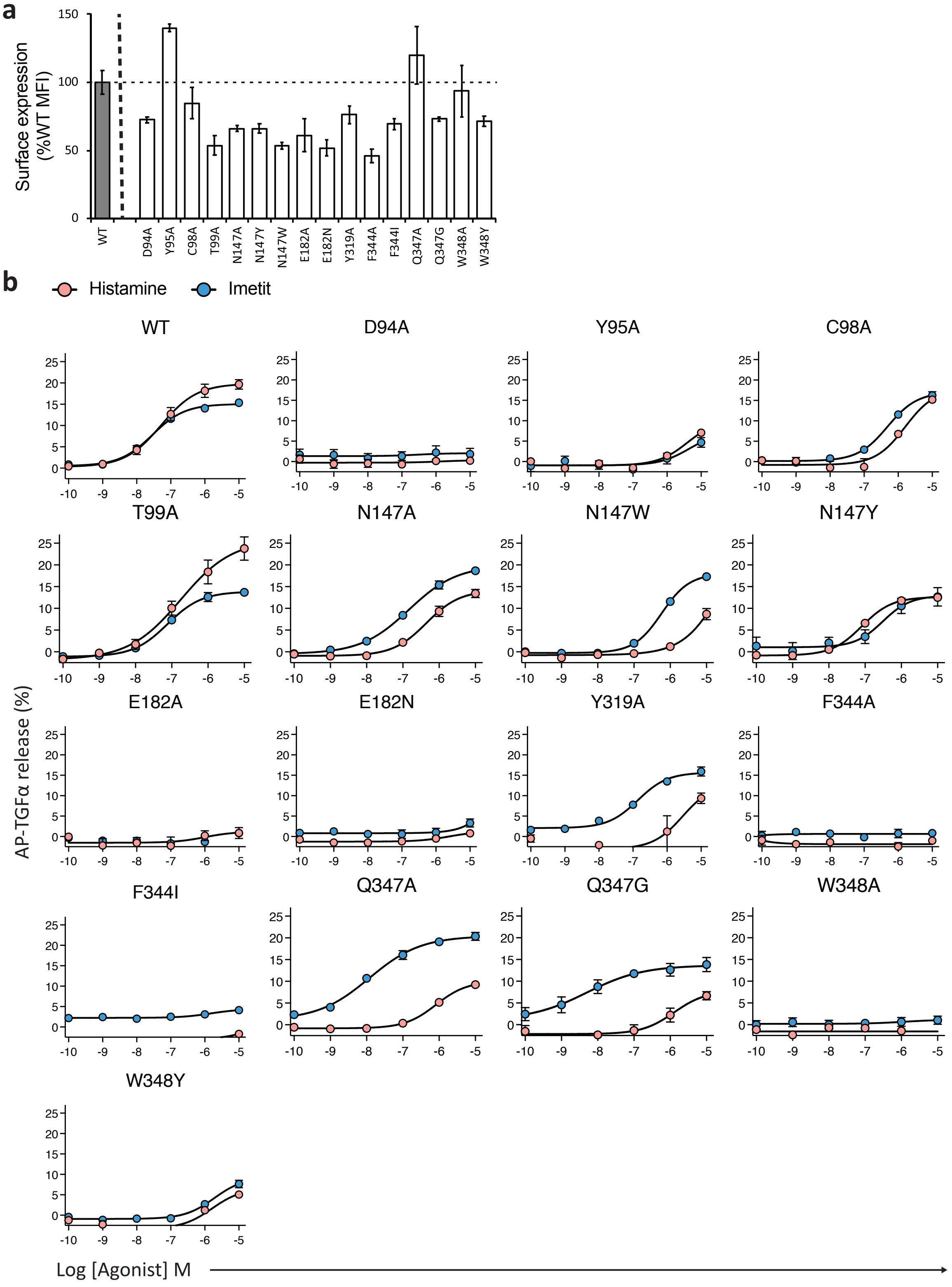

Supplementary Figure 4 | Agonist activities against the WT and mutant H_4R.

a, Surface expression of the H_4R mutants analyzed by flow cytometry. **b**, Graphical representation

of the TGFα shedding assay data for the identified H_4R WT and mutants. X-axis values are displayed as the logarithmic (Log_{10}) concentration of the respective agonist. Y-axis values are displayed as the percentage of AP-TGFα release. Dose response curves were further analyzed using the "log(agonist) vs. response-variable slope (three or four parameters)" function in GraphPad Prism 9 software (GraphPad Software Inc., San Diego, CA) (f). All data are presented as mean ± standard error of the mean of three independent experiments.

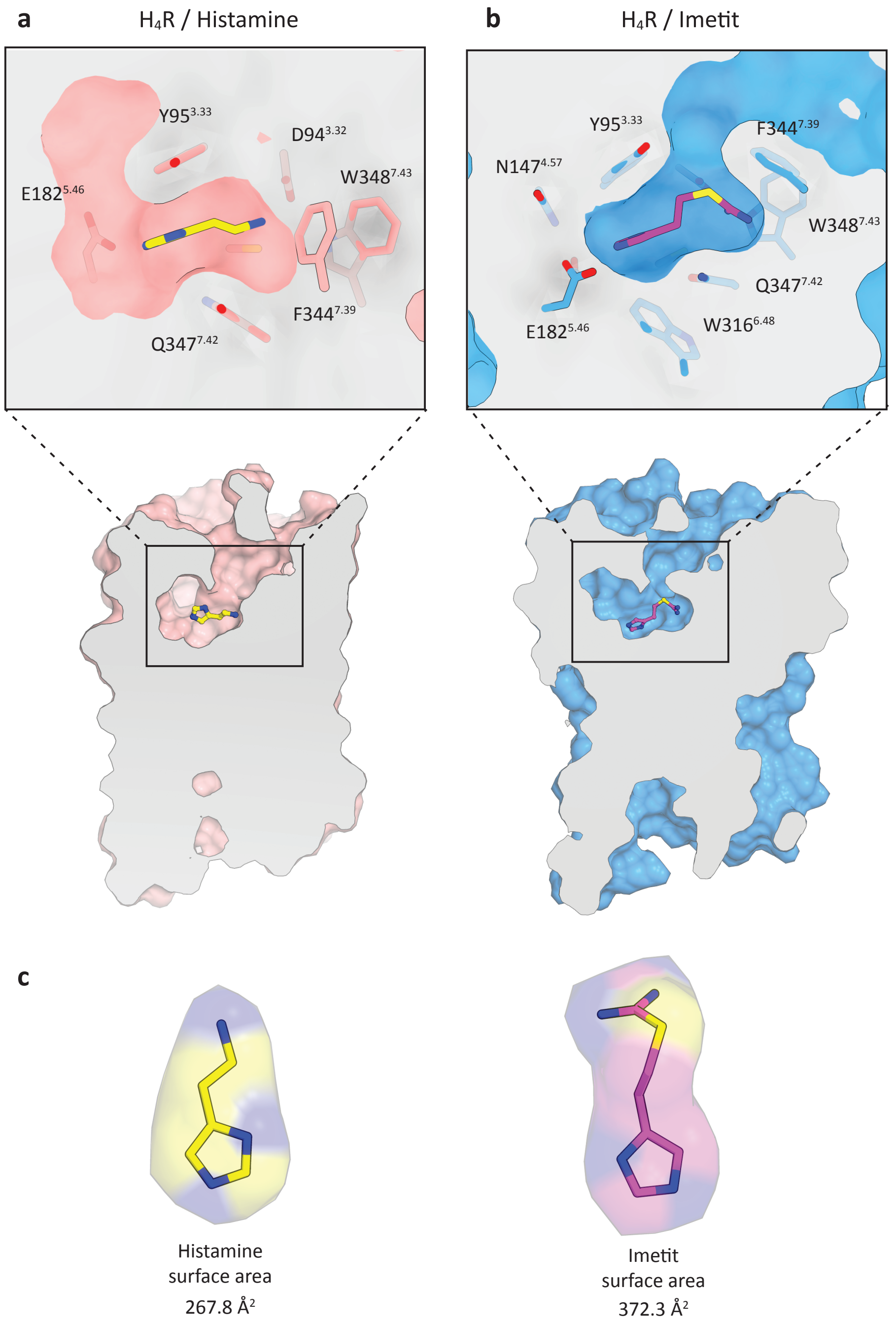

Supplementary Figure 5 | The agonist-binding pocket of H_4R_{his} and H_4R_{ime}.

a, **b**, Vertical cross-section of H_4R_{his} (**a**, pink) and H_4R_{ime} (**b**, blue). The top panels show a close-up view of the ligand-binding pocket. Structures are shown as surfaces. Ligands (histamine: yellow, imetit: magenta) and residues are shown as stick models. **c**, Comparison of the surface area of histamine (left panel) and imetit (right panel) in H_4R_{his} and H_4R_{ime}.

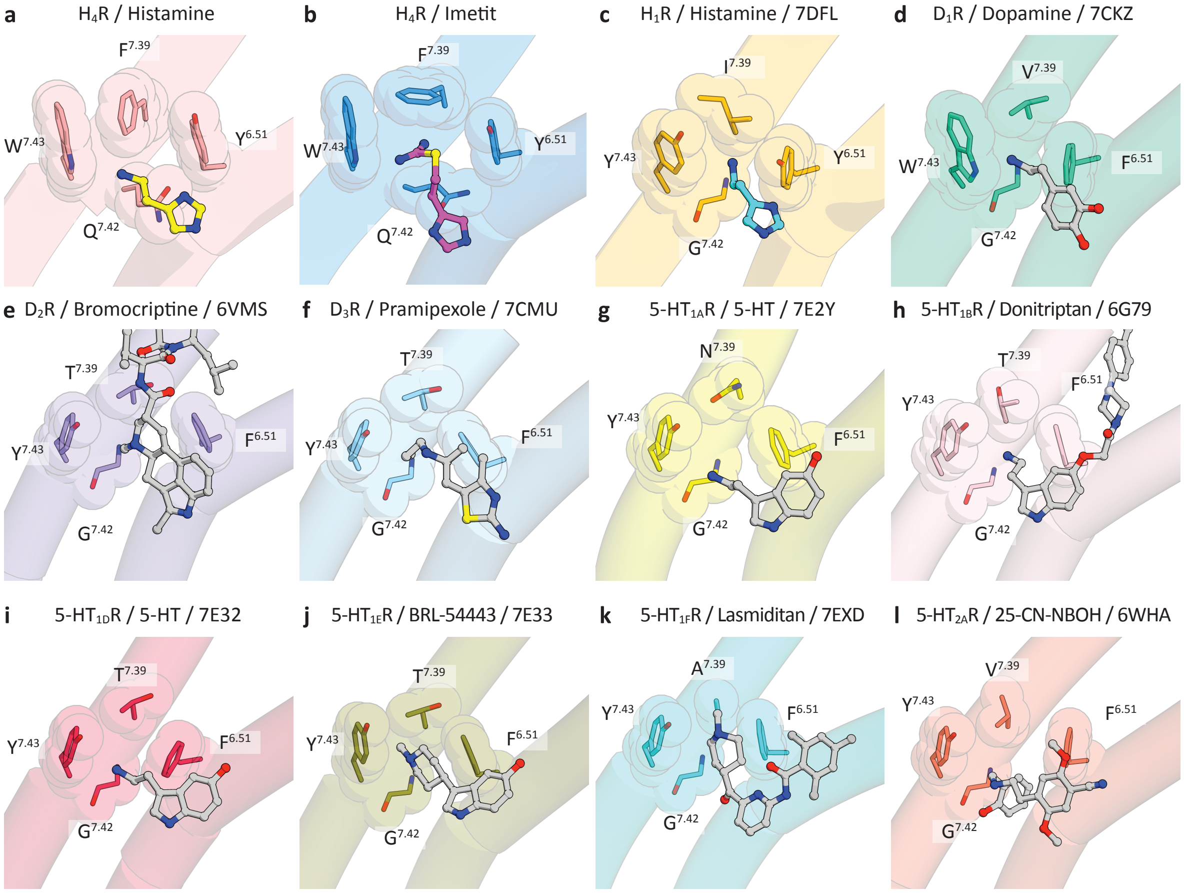

Supplementary Figure 6 | Comparison of the aromatic slot residues on the agonist-bound aminergic receptors.

Receptors and agonists are shown as cylinders and sticks, respectively. Side chains are indicated by sticks and transparent spheres. The aromatic slot on H_4R_{ime} (**b**; blue) and corresponding region for H_4R_{his} (**a**; pink), H_1R_{his} (**c**; orange), D_1R (**d**; green, PDB 7CKZ), D_2R (**e**; purple, PDB 6VMS), D_3R (**f**; light blue, PDB 7CMU), 5-$HT_{1A}R$ (**g**; yellow, PDB 7E2Y), 5-$HT_{1B}R$ (**h**; light pink, PDB 6G79), 5-$HT_{1D}R$ (**i**; red, PDB 7E32), 5-$HT_{1E}R$ (**j**; olive, PDB 7E33), 5-$HT_{1F}R$ (**k**; cyan, PDB 7EXD), and 5-$HT_{2A}R$ (**l**; tomato, PDB 6WHA).

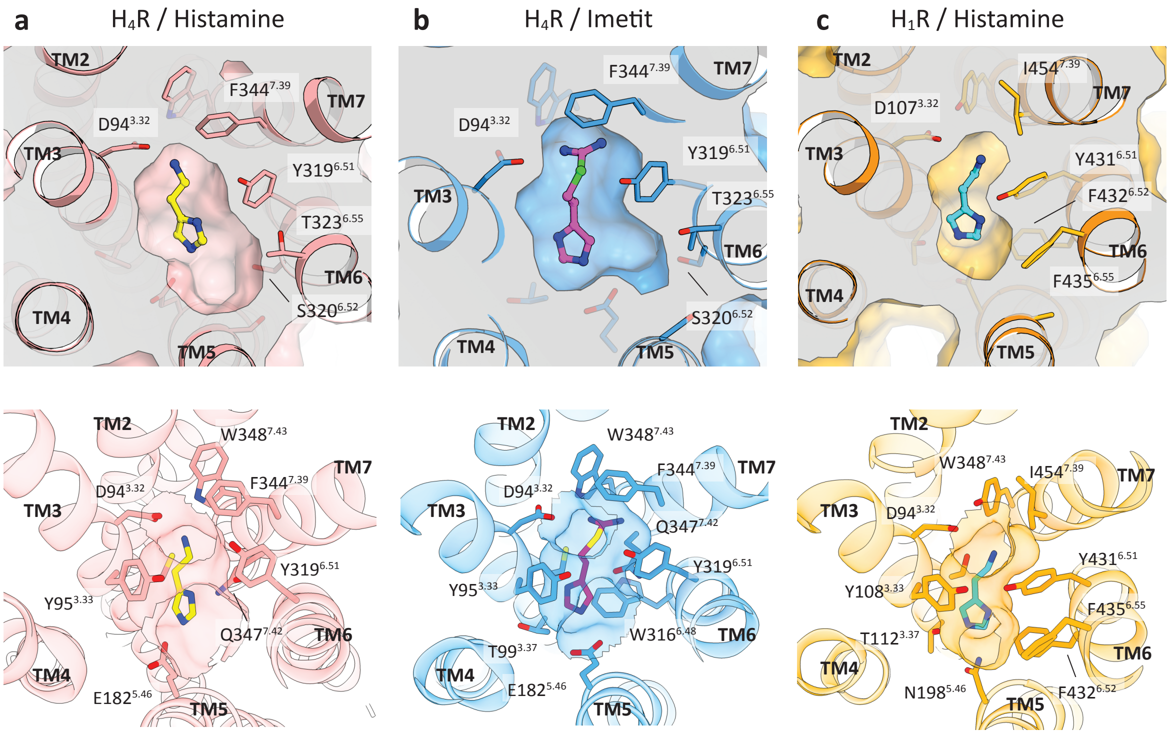

Supplementary Figure 7 | H$_4$R formed a specific sub-pocket between TM5 and 6.

a-**c**, Extracellular view of the ligand-binding pocket in H$_4$R$_{his}$ (**a**, pink), H$_4$R$_{ime}$ (**b**, blue), and H$_1$R$_{his}$ (**c**, orange). The top panels show horizontal cross-sections of each structure. The surface of the ligand-binding pocket is shown in the lower panels.

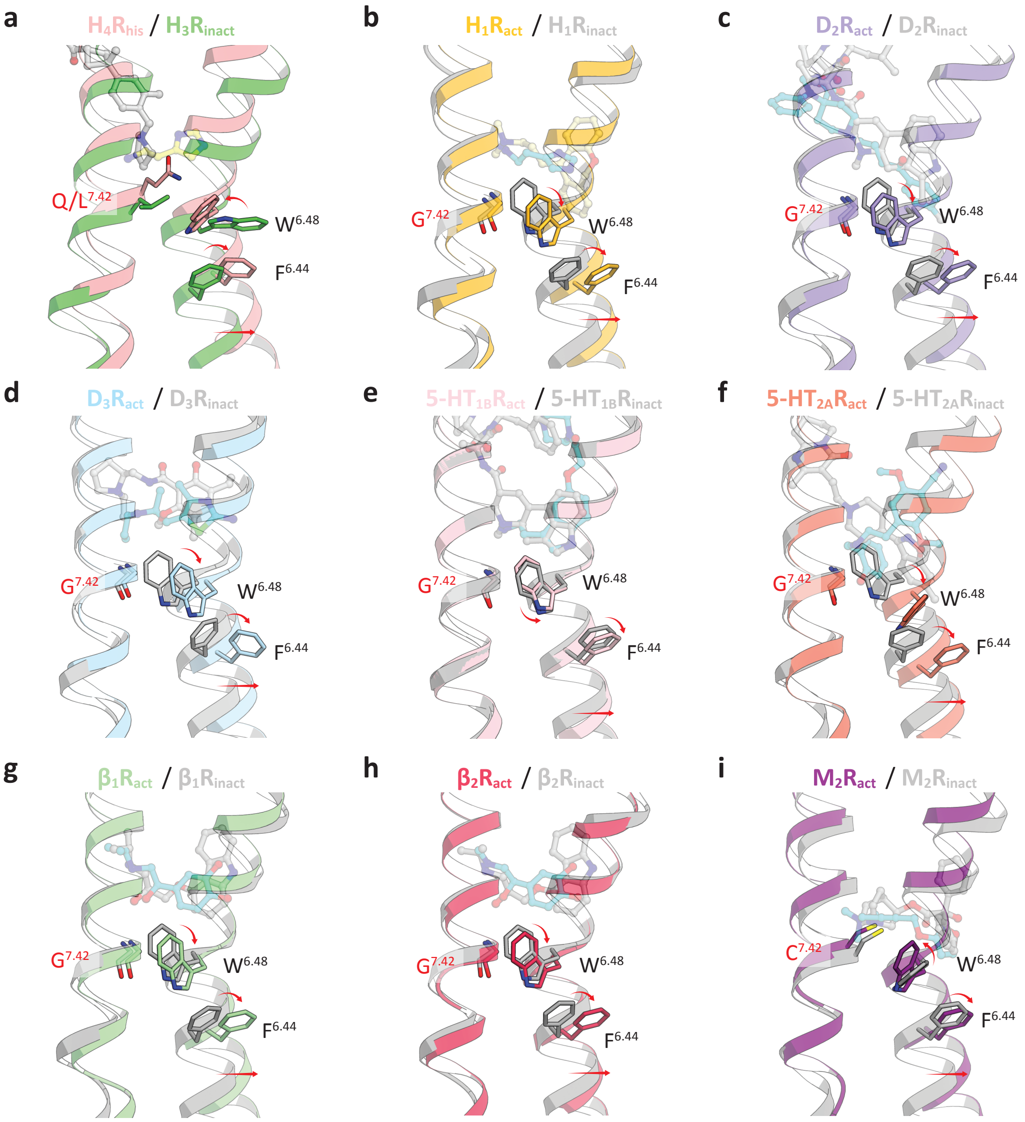

Supplementary Figure 8 | Coordination of Gln347$^{7.42}$ and the basal activity of H_4R.

a–**i**, Comparison of the peripheral structure of the residue at position 7.42. Superimposition of the inactive (gray) and active structure (each color) of aminergic receptors. The PDB IDs are shown in parentheses. (**a**) H_4R_{his}/H_3R_{inact} (7YFC, pink/7F61, green) (**b**) H_1R_{act}/H_1R_{inact} (7DFL/3RZE), (**c**) D_2R_{act}/D_2R_{inact} (6VMS/7DFP), (**d**) D_3R_{act}/D_3R_{inact} (7CMU/3PBL), (**e**) 5-$HT_{1B}R_{act}$/5-$HT_{1B}R_{inact}$ (6G79/4IAR), (**f**) 5-$HT_{2A}R_{act}$/5-$HT_{2A}R_{inact}$ (6WHA/6A93), (**g**) $\beta_1R_{act}/\beta_1R_{inact}$ (7JJO/2YCW), (**h**) $\beta_2R_{act}/\beta_2R_{inact}$ (4LDO/2RH1), (**i**) M_2R_{act}/M_2R_{inact} (6OIK/3UON). Histamine, each antagonist, and agonist are shown as yellow, gray, and cyan transparent sticks.

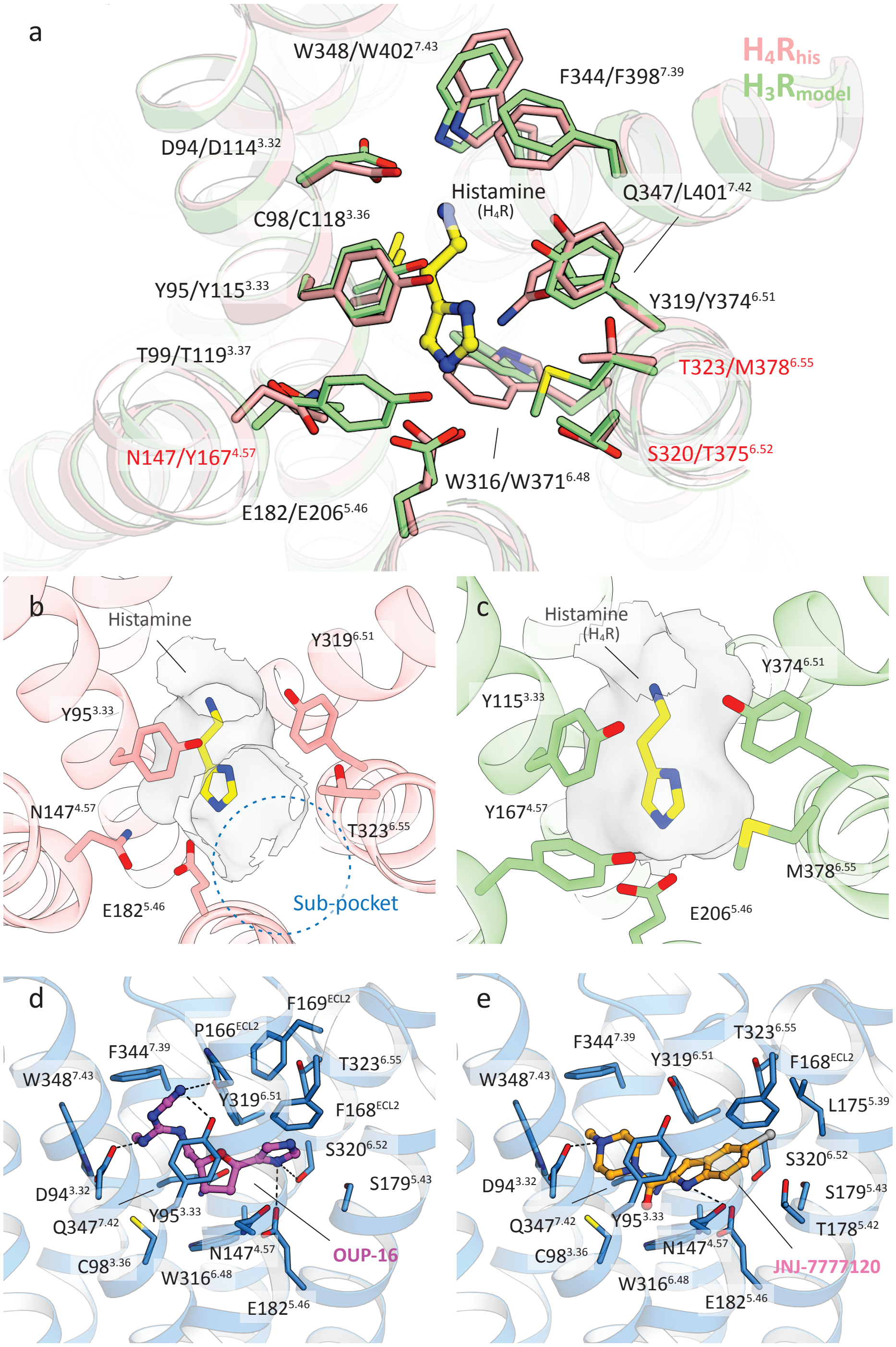

Supplementary Figure 9 | Comparison of H4R and H3R homology models demonstrated a specific sub-pocket of H4R.

a, Structural alignment of the ligand-binding pocket of H4Rhis (pink) and putative pocket of a model of H3R (green). The homology model of H3R was generated using Maestro (Schrodinger, Inc.) based on the H4Rhis structure. Histamine (yellow) and the side chains are shown as sticks. **b**, **c**,

Sub-pocket formed between TM5 and 6 in H_4R_{his} (**b**) and the corresponding region in the H_3R model (**c**). **d**, **e**, Docking simulation of H_4R specific ligands. Docking pose of the H_4R agonist OUP-16 (**d**), and the antagonist JNJ-7777120 (**e**).

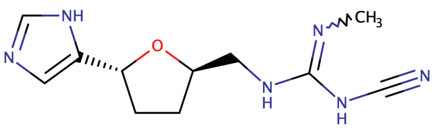

OUP-16

(H_4R-selective agonist)

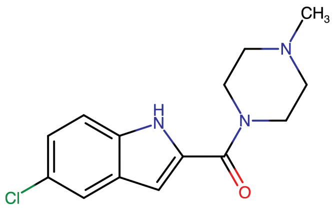

JNJ-7777120

(H_4R-selective antagonist)

Supplementary Figure 10 | Chemical structures of histamine receptor agonists and antagonists.

Supplementary Table 1 | Pharmacological parameters of the H_4R mutants against the agonists.

	Histamine				Imetit			
	pEC_{50} mean ± SEM	Emax (%) mean ± SEM	EC_{50} (nM)	fold of change	pEC_{50} mean ± SEM	Emax (%) mean ± SEM	EC_{50} (nM)	fold of change
WT	7.12 ± 0.08	19.42 ± 1.15	55.4	1	7.53 ± 0.06	14.93± 1.19	29.9	1
D94A	ND	ND	ND	+++	ND	ND	ND	+++
Y95A	ND	ND	ND	+++	ND	ND	ND	+++
C98A	ND	ND	ND	+++	6.32 ± 0.03*	17.06 ± 1.72	486.2	16.3
T99A	6.93 ± 0.05	22.78 ± 4.74	134.9	2.4	7.12 ± 0.01	13.72 ± 1.67	76.0	2.5
N147A	6.15 ± 0.13	14.11 ± 1.55	510.3	9.2	6.94 ± 0.04*	17.18 ± 0.52	116.1	3.9
N147W	ND	ND	ND	+++	6.22 ± 0.01*	18.45 ± 1.29	602.8	20.2
N147Y	7.09 ± 0.05	12.71 ± 0.59	82.9	1.5	6.94 ± 0.04	11.10 ± 1.47	319.4	10.7
E182A	ND	ND	ND	+++	ND	ND	ND	+++
E182N	ND	ND	ND	+++	ND	ND	ND	+++
Y319A	ND	ND	ND	+++	6.85 ± 0.16	15.81 ± 1.58	159.4	5.3
F344A	ND	ND	ND	+++	ND	ND	ND	+++
F344I	ND	ND	ND	+++	ND	ND	ND	+++
Q347A	6.06 ± 0.04*	10.06 ± 0.39	826.9	14.9	7.91 ± 0.07*	19.34 ± 1.10	12.7	0.4
Q347G	6.47 ± 0.38	8.80 ± 2.03	2570.2	46.4	8.33 ± 0.24	12.90 ± 1.22	6.2	0.2
W348A	ND	ND	ND	+++	ND	ND	ND	+++
W348Y	ND	ND	ND	+++	ND	ND	ND	+++

ND, EC_{50} or Emax values cannot be detected due to their low signal; +++, Exceedingly high fold of change due to undetectable or very low agonist activity; All data are presented as mean ± SEM from three independent experiments performed in triplicate. *$p < 0.05$, one-way ANOVA, followed by Dunnett's post-test.

Supplementary Table 2 | List of primers sequences for site-direct mutagenesis studies.

Oligonucleotides primer	Forward	Reverse
D94A	ACCACCGCTTACCTGCTGTGCACCGCT	CAGGTAAGCGGTGGTCAGCCAGAAAAC
Y95A	ACCGACGCTCTGCTGTGCACCGCTTCC	CAGCAGAGCGTCGGTGGTCAGCCAGAA
C98A	CTGCTGGCTACCGCTTCCGTGTACAAC	AGCGGTAGCCAGCAGGTAGTCGGTGGT
T99A	CTGTGCGCTGCTTCCGTGTACAACATC	GGAAGCAGCGCACAGCAGGTAGTCGGT
N147A	TTCCTGGTCGCTGGTCCCATGATCCTGGTG	CATGGGACCAGCGACCAGGAAAGCCAGCAC
N147W	CTGGTCTGGGGTCCCATGATCCTGGTG	GGGACCCCAGACCAGGAAAGCCAGCAC
N147Y	TTCCTGGTCTATGGTCCCATGATCCTGGTG	CATGGGACCATAGACCAGGAAAGCCAGCAC
E182A	TTCTTGGCTTTCGTGATCCCCGTGATC	CACGAAAGCCAAGAACGATGTGATAGC
E182N	TCGTTCTTGAATTTCGTGATCCCCGTGATC	GATCACGAAATTCAAGAACGATGTGATAGC
Y319A	TGGGCTCCCGCTTCCCTGTTCACCATCGTC	GAACAGGGAAGCGGGAGCCCAGCAAACGGC
F344A	AGGATCGCCGCTTGGCTGCAGTGGTTCAAC	CTGCAGCCAAGCGGCGATCCTGTACCACAC
F344I	ATCGCCATTTGGCTGCAGTGGTTCAAC	CAGCCAAATGGCGATCCTGTACCACAC
Q347A	TGGCTGGCTTGGTTCAACTCCTTCGTG	GAACCAAGCCAGCCAGAAGGCGATCCT
Q347G	TTCTGGCTGGGTTGGTTCAACTCCTTCGTG	GTTGAACCAACCCAGCCAGAAGGCGATCCT
W348A	CTGCAGGCTTTCAACTCCTTCGTGAAC	GTTGAAAGCCTGCAGCCAGAAGGCGAT
W348Y	CTGCAGTATTTCAACTCCTTCGTGAAC	GTTGAAATACTGCAGCCAGAAGGCGAT
